# Aptamers based sensing of pregnancy associated glycoproteins (PAG) of bovine for early pregnancy detection

**DOI:** 10.1038/s41598-021-02551-1

**Published:** 2021-12-01

**Authors:** Munna Lal Yadav, Abhishek Parashar, Nimai Charan Mahanandia, Vanya Bhushan, Sudarshan Kumar, Ashok Kumar Mohanty

**Affiliations:** 1 Translational Health Science and Technology Institute (THSTI), Faridabad, India; 2 Animal Biotechnology Centre, National Dairy Research Institute (NDRI), Karnal, 132001 India; 3 Indian Agricultural Research Institute (IARI), Pusa, New Delhi, Delhi 110012 India; 4 Indian Veterinary Research Institute (IVRI), Mukteswar, 263138 India

**Keywords:** Biotechnology, Molecular biology

## Abstract

Tosyl activated magnetic beads were used for aptamer selection against PAG- 7 and 18 proteins of bovine origin. PAG proteins were immobilized on beads with further addition of biotin tagged aptamer library. The recognition of aptamers with PAG was identified by ST-HRP based approach which was colorimetric in nature. The selected aptamers were sequenced and at the same time several new aptamers were identified. Later M-fold structure and G-quadruplex score of aptamers were analyzed for their selection. Those aptamers having high G value and complex structure were chosen. In dot blot assay, aptamers recognized PAG protein in an animal after 42 days of artificial insemination which later given birth to a healthy calf. Further the cross reactivity with serum of 0th day animal (post AI) or with non pregnant animal serum was minimal. Aptamers have also shown interaction with PAG protein of buffalo origin. These selected aptamers have commercial application especially in development of biosensors for early detection of pregnancy in bovine.

## Introduction

Early pregnancy detection in bovine is key to better reproductive management in livestock^1^. There are several methods by which detection can be performed such as rectal palpation^2^, ultra sound^3^ etc. These all methods have their own pros and cons. Apart from these manual methods, a recent and more reliable technique involving pregnancy associated markers that are exclusively expressed during pregnancy, such as pregnancy associated glycoprotein (PAG)^4^, progesterone hormone^5^ can be better alternatives to manual methods. ELISA based kits are available against PAG 1 protein^6^ and progesterone^7^. In an unpublished work antibody has also been generated against PAG-7 & PAG-18 proteins that are abundantly present during bovine pregnancy. ELISA and lateral flow based detection methods are currently in developing stages against these proteins.

Aptamers are new class of synthetic molecules that are widely used for detection and therapeutic purposes in recent years^8^ and have certain advantages over antibodies such as low cost, animal free production, no batch to batch variation etc.^9^. The method of aptamer selection is based on SELEX (Systematic evolution of ligands by exponential enrichment) which is an iterative method and is generally performed in ten to fifteen cycles^10,11^.

In past few years SELEX method has been modified to enhance the aptamer selection process. Single step or non-SELEX method has been tried in selecting aptamer against several targets. In one of the method, florescence tagged aptamer was incubated with snake venom (α-bungarotoxin) which was immobilized on cover slip. Only those aptamers which were bound to snake venome was taken for further analysis and was found useful in detection of venom^12^. In MonoLEX method, target is attached to resin of column and aptamers were passed through the column. Those sequences having affinity with target retain in the column which was later amplified by cutting the column. Researcher selected aptamers against Vaccinia virus by this method^13^. X aptamer is another example which is selected by single step selection method^14^.

The proposed work for aptamer selection is non-SELEX based method and is fast & reliable. Further it is colorimetric in nature. The method uses magnetic bead based approach where PAG protein is immobilized on beads and biotin labeled aptamers are added on it. The tube containing beads appears blue in color after adding TMB which is based on streptavidin-HRP interaction with biotin sequence. Only those tubes are selected that give intense blue color in shortest period of time. PCR was performed for selected beads to get amplified product of aptamers. Amplicon sequencing of PCR product gave information regarding aptamer sequences. The method is novel in the sense that it provides a visual based SELEX selection method which is not possible with conventional SELEX. Further only more interactive aptamers (on the basis of color intensity) are chosen by this method which otherwise would be a time consuming and labor intensive with conventional method.

## Material and method

### Selection of aptamers by non-SELEX method

20 μl of magnetic beads having Tosyl functional group (Dynabeads M280 tosyl activated beads, code no. 14203, purchased from Invitrogen, USA) were taken in five separate tubes and 10 μg of peptide sequence (KIPLRRVKTMRKTLSGKN) of PAG-18 was immobilized in on all tubes containing beads except first tube which was marked as control. The immobilization was done in presence of 150 μl borate buffer (0.1 M), pH 9.5. Further, 100 μl of 3.0 M ammonium sulphate (prepared in borate buffer, pH 9.5) was added to the mixture. The mixture was incubated at 37 °C for 22 h. The immobilized beads were blocked in 1.0 M tris for 3 h. Further the beads were blocked by adding 2% of BSA for 6 h at RT. Similarly, immobilized beads in control tube were also blocked in same fashion. After blocking, 50 ul of 1 × PBS was added to all tubes and were incubated overnight at 4 °C.

Next day 1.0 μM of 50 ul biotin labeled aptamer library (TAGGGAAGAGAAGGACATATGAT-N40-TTGACTAGTACATGACCACTTGA, Integrated DNA Technologies, Belgium) was taken and dissolved in 100 μl of 1 × PBS. 0.2 μl of magnesium chloride (1.0 M) was further added to the mixture. The mixture was snap cooled (heating at 96 °C for 15 min and quick cooling in ice for 5 min) and 30 μl of each of aptamer mixture was added to all tubes at room temperature for 1 h. After incubation, supernatant was discarded and a total of five washing with 1 × PBS was given to remove unbound aptamers. Streptavidin conjugated with horseradish peroxidase (ST-HRP, code-N100, purchased from Thermo Fisher, USA) were diluted to 1/30,000 times and was added to all the tubes for 20 min duration and was later washed with 1 × PBS before adding TMB (code-T0440, purchased from Sigma, Germany) solution for color development.

The tube that has given most intense blue color in shortest period of time was picked and washed with water. After washing, 2.0 μl of beads were taken in four PCR tubes and amplification was performed at following temperature: initial heating at 95 °C for 3 min 28 cycles of 95 °C for 30 s., 50 °C for 30 s, 72 °C for 30 s. and final extension of 72 °C for 5 min with cool down at 4 °C and checked at 2% agarose gel. The PCR product was purified and sent for amlicon sequencing. Similar protocol for aptamer selection was also followed for peptide sequence (KDSRGHCYTTFKEKRVRRS) of PAG-7 protein.

### Sequence and structural analysis of selected aptamers

The sequences of PAG-7 and 18 aptamers generated by non-SELEX method were further studied for secondary structure analysis by online M-fold program. The predicted secondary structure based on lowest to highest free energy gives information regarding stem/loop structure of aptamers that might be involved in interaction with peptides. The G-quadruplex analysis of sequences was performed by using QGRS mapper.

### Homology Modeling of PAG-7 and PAG-18

The amino acid sequences of Pregnancy Associated Glycoprotein 7 (PAG-7) and Pregnancy Associated Glycoprotein 18 (PAG-18) were retrieved from the protein sequence (UniPortKb) (https://www.uniprot.org) database. BLASTp tool of NCBI was used for identifying a suitable template for the computational modeling of PAG-7 and PAG-18. The secondary structures of PAG-7 and PAG-18 were predicted using PSIPRED server. The three dimensional (3-D) model of PAG-7 and PAG-18 was constructed using the homology modeling tool MODELLER v9.24. The best structure was selected according to the lowest discrete optimized protein energy (DOPE) score. After that, Proteins structure was subjected to energy minimization using GROMACS. Further, the model quality and validation was done through PROCHECK and ERRAT software’s. Further, Energy profile characterization was performed using ProSA.

### Molecular docking

AutoDock v4.2 (http://autodock.scripps.edu/) tool was used for molecular docking analysis. The six aptamers were docked against the PAG-7 and four aptamers were docked against the PAG-18. In AutoDock tool, the polar hydrogen was added into the PAG-7 and PAG-18 proteins and Kollman charges were assigned for optimization of proteins. The Geister partial charges were applied on aptamers and non-polar hydrogens were merged to the aptamers. Then, the structures of the proteins and aptamers were saved in “.pdbqt” format that was used later for docking calculation. A grid maps was prepared for the docking calculation. In the PAG-7 protein, a 3-D grid box with dimensions X = 72, Y = 60, and Z = 72 was created with grid-point center X = − 0.167, Y = 0.194, Z = − 4.639 while the grid spacing was set to 1.000 ÅÅ. For PAG-18, a 3-D grid box with dimensions X = 68, Y = 60, and Z = 58 was created with grid-point center X = − 2.806, Y = − 1.528, Z = − 9.306 and the grid spacing was set to 1.000 Å. The docking conformation was analyzed by using the Lamarckian Genetic Algorithm (LGA). In the docking process, a maximum of 50 conformers was considered for each aptamers and the results were ranked based on their binding energy score and top aptamer with lowest binding energy score was carried out forward for further studies.

### Detection of PAG proteins by aptamers

#### Use of serum sample obtained from blood of bovine

5.0 μl of streptavidin coated gold nanoparticles (ST-GNP), (Cytodiagnostics, cat. no. AC-40-04-05, conc. 0.15 mg/ml) was taken and mixed with in 20 μl of 1 × PBS. Gold nanoparticle solution was further mixed with 25 μl of 1 μM of biotin labeled PAG7_2 aptamer (Sigma). The incubation was done for an hour at RT. Tube was centrifuged at 12,000×*g* for 5 min. The process was repeated thrice and each time unbound aptamers were discarded by removing the supernatant and finally 20 μl of 1 × PBS was added. Now 10 μl of serum solution extracted from blood of 0th day animal (cattle just before the artificial insemination was considered as 0th day animal) was taken and spotted at nitrocellulose membrane (NC), (pore size 0.45 μm) on spot no. 2 on membrane surface. Spot no. 1 on membrane was spotted with 10 μl solution of serum from an animal, 42 days post AI. The membrane was dried for an hour at RT and blocked in 5% BSA solution for 2 h. After the incubation, 5 μl of aptamer conjugated GNP was added on all the three spots on the membrane namely spot no. 1 and 2 while spot no.3 was the untreated (control) spot on membrane. The membrane was incubated for 45 min and then washed thrice by 1 × PBS for color development and observation.

In separate experiment, four spots were made on two strips of nitrocellulose membrane of 0.45 μm pore size. Spot no. 1 was left untreated while 4.0 μl solution of serum of an animal already passed 42 days after an AI was added at spot no. 2. 4.0 μl of serum solution extracted from blood of animal (0 day animal) was spotted at spot no. 3 while spot no. 4 had 4.0 μl of 10% BSA solution. The membrane was dried for an hour at RT and blocked in 5% BSA solution for 2 h. After the incubation, 5.0 μl of 1.0 μM biotin labeled aptamers (PAG7_2 and PAG7_29) was spotted at strip 1 and 2 respectively for 45 min at RT. Subsequently, strips were washed and immersed in streptavidin conjugated to HRP (5 μl of 1.25 mg/ml was dissolved in 10 ml 1 × PBS) for 30 min. A brief washing was again given to strips in 1 × PBS, followed by addition of 5.0 μl solution of 3,3′-Diamino benzidine (DAB purchased from GeNei, India) on each spot in dark. The brown color was observed after 15 min. Subsequently strips were also exposed to TMB for five minutes for enhanced blue color formation.

In another experiment carried with same gold nanoparticle, biotin labeled sequence PAG7_29 was coated on ST-GNP as mentioned earlier. 10 μl of serum solution extracted from animal blood (0th day animal) was taken and spotted on nitrocellulose membrane (spot no. 2) of pore size 0.45 μm which will also be considered as control here. Spot no. 1 was spotted with 10 ul serum solution of an animal, 42nd days post AI. The membrane was dried for an hour at RT and blocked in 5% BSA solution for 2 h. After the incubation 5.0 μl of aptamer conjugated GNP was added at spot no. 1 and 2. The membrane was incubated for 45 min and then washed thrice by 1 × PBS for color development and observation. In same manner PAG7_47, PAG7_62, PAG7_76 & PAG 18_91 were also tested for PAG recognition.

#### Use of serum sample obtained from blood of Water buffalo

PAG18_21 and PAG7_29 aptamers were also tested to detect PAG proteins in buffalo serum: from a buffalo 100 days post AI and from a buffalo that has just given birth to a calf (1 day post calving) by using ST-GNP approached as mentioned earlier. The intensity of interaction was further enhanced by using silver enhancer (Cytodiagnostics, cat no. SR-01–02).

In another experiment working with same ST-GNP approach, PAG18_8 aptamer was tested against serum of 0th day animal (n = 2) for detection of PAG protein. Further, aptamer detection test for PAG was done against serum samples from 28th, 35th and 42nd days of animal post AI. In similar manner aptamer PAG18_8 was tested for its interaction to PAG of an animal that had passed 28 days of AI in time dependent manner. The time given for interaction was set at 5, 10 and 20 min. respectively. Further on control spot no PAG was added, only membrane was incubated with aptamer. The condition of experiment was similar to what was followed in gold nanoparticle based detection method section. In another experiment on nylon membrane (Roche Diagnostics, cat. no. 11209299001) three spots were made by pencil. On spot no. 1 and 2, 5.0 μl of 1.0 μM aptamer PAG7_47 that contained amino group at 5’ end was spotted. The membrane was incubated for 5 min in UV light at 254 nm and subsequently blocked in 5% SDS for 12 h at RT. Next day, 2.0 μl of serum sample from 0 and 28th day animal post AI was spotted on spot no. 1 and 2 respectively for 20 min. The membrane was briefly washed in 1 × PBS. Afterwards 5’ biotin labelled sequence (which was complementary of random region of aptamer 47) was immobilized on ST-GNP (as mentioned above). 5.0 μl GNP conjugated aptamer 47 was added to each spots for 10 min. The membrane was washed and dried for colour development and observation.

In competitive color based experiment, 10 μl of serum solution of an animal (0th day animal) was spotted on two different strips of nitrocellulose membrane (pore size 0.45 μm) on spot no. 3 and 4 respectively. While on spot no. 1 and 2, 10 μl serum solution from a pregnant animal (42nd day post AI) was spotted. The membranes were dried for an hour at RT and blocked with 5% BSA solution for 2 h. 5.0 μl of streptavidin coated gold nanoparticles (ST-GNP) was taken in four tubes and mixed with 20 μl of 1 × PBS. The mixture was further mixed with 25 μl of 1 uM of biotin labeled PAG18_8R, PAG18_91R, PAG7_29R and PAG7_47R respectively. All the tubes were incubated for an hour at RT. Tubes were centrifuged at 12,000×*g* for 5 min. The process was repeated thrice and each time unbound aptamers were discarded by removing supernatant and in the final step 15 μl of 1 × PBS was added to all the tubes. 5 μl of aptamer (Seq. PAG18_91R) conjugated GNP was spotted at spot no. 1 and 3 while Seq. PAG18_8R was spotted on spot no. 2 and 4 of first strip of membrane. On second membrane strip, Seq. PAG7_29R was spotted on spot no. 1 and 3 while Seq. PAG7_47R on spot no. 2 and 4. Both the membrane strips were incubated 15 min. Subsequent washing was given with 1 × PBS so as to remove any unbound aptamers. In the final step, membrane strips were air dried and colour change was observed.

In the following experiment, nitrocellulose membrane (pore size 0.45 µm) was taken and three spots were made on it. 4.0 μl of serum sample from an animal (0th day post AI) was taken and spotted at spot no. 2. Spot no. 1 on membrane was spotted with 4.0 μl serum sample from an animal, 42nd day post AI while 4.0 μl of 10% BSA solution was taken as control and was spotted at spot no. 3. The membrane was dried for an hour at RT and blocked with 5% BSA solution for 2 h. After the incubation 5.0 μl of 1.0 μM aptamer (PAG18_91), conjugated to biotin at 5’ end was added on spot no. 1, 2 & 3 and was incubated for 45 min at RT. The membrane was washed with 1 × PBS. Streptavidin conjugated to HRP was prepared (5 μl of 1.25 mg/ml was dissolved in 10 ml 1 × PBS) and membrane strip was immersed in that solution for 30 min. A brief washing was given to membrane with 1 × PBS and subsequently 5.0 μl solution of 3,3′-Diamino benzidine (DAB) was added on each spot in complete darkness. Color change was observed after 15 min. The same set up of experiment was also performed by using ECL (enhanced chemiluminescence purchased from Bio-Rad Company) for PAG18_91. Test was also carried out to see aptamer (PAG18_91) interaction with PAG-18 peptide, recombinant PAG-7 and 18 proteins and BCM-7 (YPFPGPI) peptide.

Aptamer PAG7_47 interaction was checked with synthetic PAG-7 protein peptide as well as with natural PAG-7 protein present in pregnant sample by using DAB. In ECL experiment also (by maintaining same condition as mentioned above) PAG7_47 was used to see aptamer interaction with PAG protein of pregnant animal’s serum sample. Further by using same above mentioned protocol, 4.0 μl of recombinant PAG-7 and 18 proteins were spotted at nitrocellulose membrane (spot no. 1 and 3). While at spot no. 2, same volume of 10% BSA was spotted. In second strip of NC, synthetic PAG-7 protein peptide, BSA & BCM-7 peptides were spotted at spot no. 1, 2 and 3 respectively. The strips of membrane were dried for an hour at RT and blocked in 5% BSA solution for 2 h. After that 5.0 μl of biotin labeled 1.0 μM aptamer (PAG7_47) was added to all spots for 45 min. The strips were washed and streptavidin conjugated to HRP was added for 30 min. A brief washing was given to membrane with PBS and ECL was used for color development.

### Ethical clearance

From the institute animal ethical committee of National Dairy Research Institute (IAEC- NDRI), approval was taken for blood sampling of animals vide letter no. 41-IAEC-18-50, Dated 27.1.2018. Further, all the experiments performed under this work were carried out in compliance with the ARRIVE guidelines & accordance to institutional guidelines of National Dairy Research Institute.

## Result

**Figure 1:**
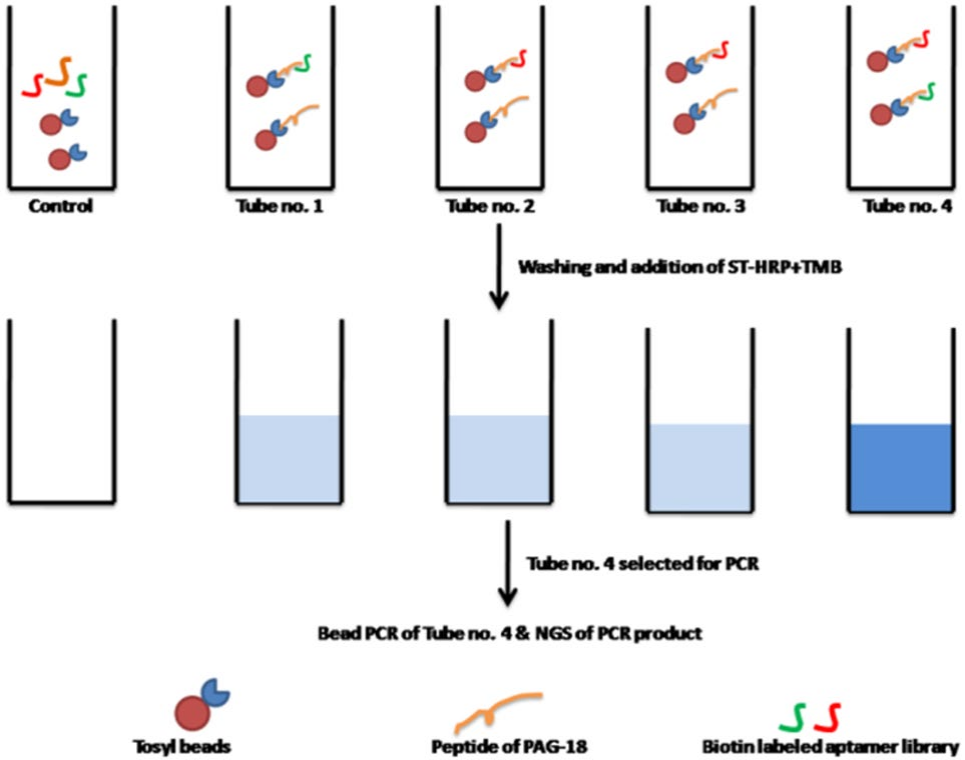
Diagrammatic sketch of aptamer selection.

**Figure 2:**
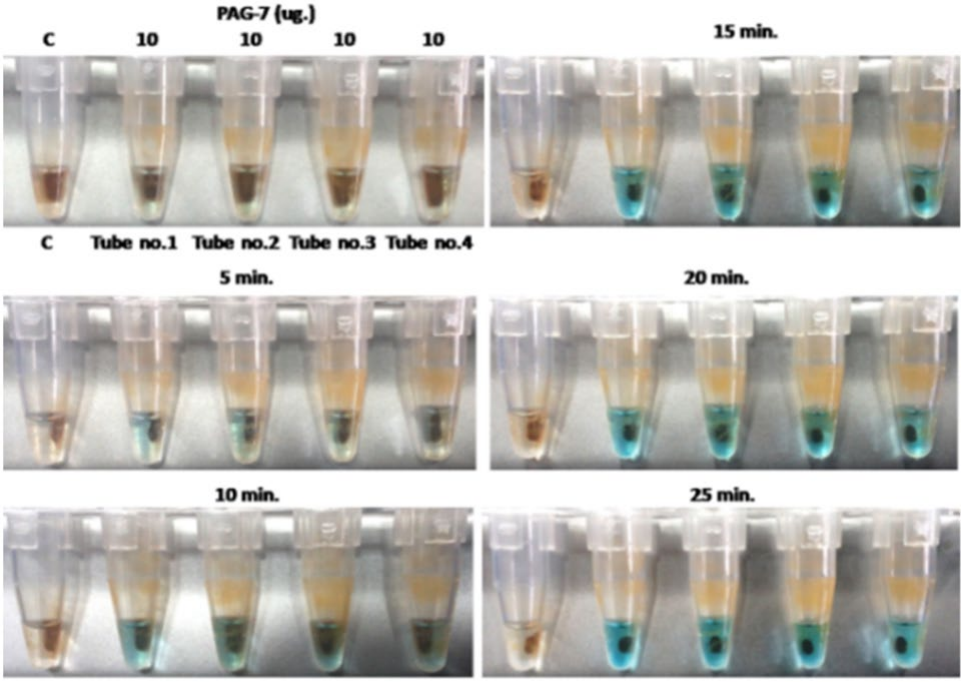
Magnetic bead based colorimetric method for aptamer selection against PAG-7 protein.

**Figure 3:**
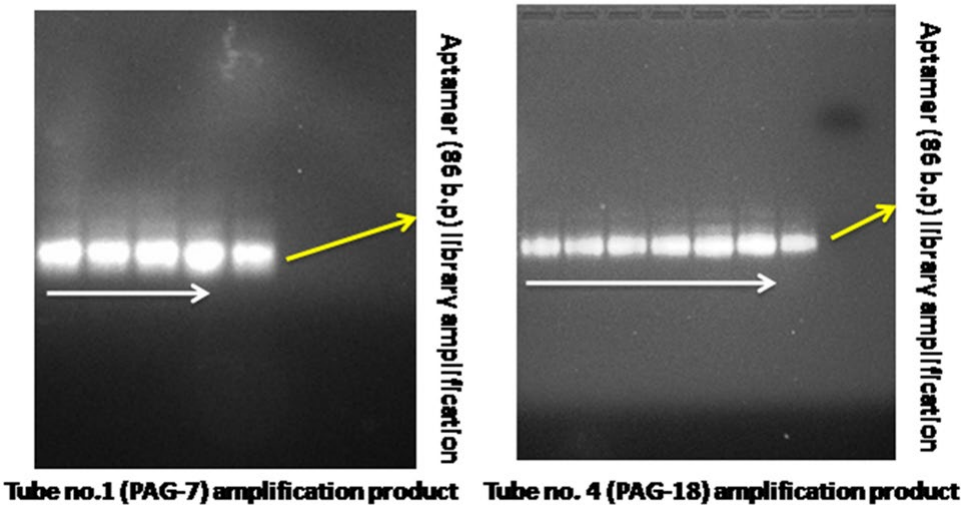
Agarose gel run of amplified aptamers, selected against PAG proteins.

At the end of non-SELEX selection process, the tubes (tube no. 1 for PAG-7 and tube no. 4 of PAG-18 proteins) which has given intense blue color in quick time was used for aptamer amplification by PCR (Figs. 1 and 2). The presence of correct aptamer band on gel was identified by matching band position of aptamer library of 86 bases which was also amplified at same PCR condition (Fig. 3).

After amplification the PCR product was sent for amplicon sequencing. Several new aptamers were identified (Table 1).

**Table 1:** Aptamers sequence used in experiment.

Name	Sequence (5′–3′ direction)		
	Constant region (C)	Random region (R)	Constant region (C)
PAG7_2	TAGGGAAGAGAAGGACATATGAT	GGGTGAGCCGGACGGGGGGCTGGCAAGGGACGGGGGGGCTG	TTGACTAGTACATGACCACTTGA
PAG7_29	TAGGGAAGAGAAGGACATATGAT	TCGTGCAGCTATTCGCTGCTCACGTCCGTCTGTTTCCTGC	TTGACTAGTACATGACCACTTGA
PAG7_29R		TCGTGCAGCTATTCGCTGCTCACGTCCGTCTGTTTCCTGC	
PAG7_47	TAGGGAAGAGAAGGACATATGAT	GCGTATTACCTAGTTTGAGTCCCATGAAACGATGCACTGG	TTGACTAGTACATGACCACTTGA
PAG7_47R		GCGTATTACCTAGTTTGAGTCCCATGAAACGATGCACTGG	
PAG7_57	TAGGGAAGAGAAGGACATATGAT	AAGTGAACATGACACTGGAACATCCGAGCGCAAATTAAAC	TTGACTAGTACATGACCACTTGA
PAG7_76	TAGGGAAGAGAAGGACATATGAT	TCATCCCTAGCGGGTCGGGCGGCGCTCGCGGCCCAGGGTA	TTGACTAGTACATGACCACTTGA
PAG18_8	TAGGGAAGAGAAGGACATATGAT	ATGGTCCGTAGGTCTCTGGGATGTTTTTTGTCGGACTTTA	TTGACTAGTACATGACCACTTGA
PAG18_8R		ATGGTCCGTAGGTCTCTGGGATGTTTTTTGTCGGACTTTA	
PAG18_19	TAGGGAAGAGAAGGACATATGAT	GTAACGGGCGGCAAGGGTTAGGTGCGGGTTCCGCGGGCGG	TTGACTAGTACATGACCACTTGA
PAG18_21	TAGGGAAGAGAAGGACATATGAT	GATAGTCAAGCGCGGGCAATTCGCTTGTTACACTTCCCAG	TTGACTAGTACATGACCACTTGA
PAG18_62	TAGGGAAGAGAAGGACATATGAT	CCCGCTGGTTCGCTCGTGGTAAGGTACCTAGGTCGAATGA	TTGACTAGTACATGACCACTTGA
PAG18_91	TAGGGAAGAGAAGGACATATGAT	TATCATTCCTTTTAGTGGAGACACACGCATCTCGTGTGCT	TTGACTAGTACATGACCACTTGA
PAG18_91R		TATCATTCCTTTTAGTGGAGACACACGCATCTCGTGTGCT	

**Figure 4:**
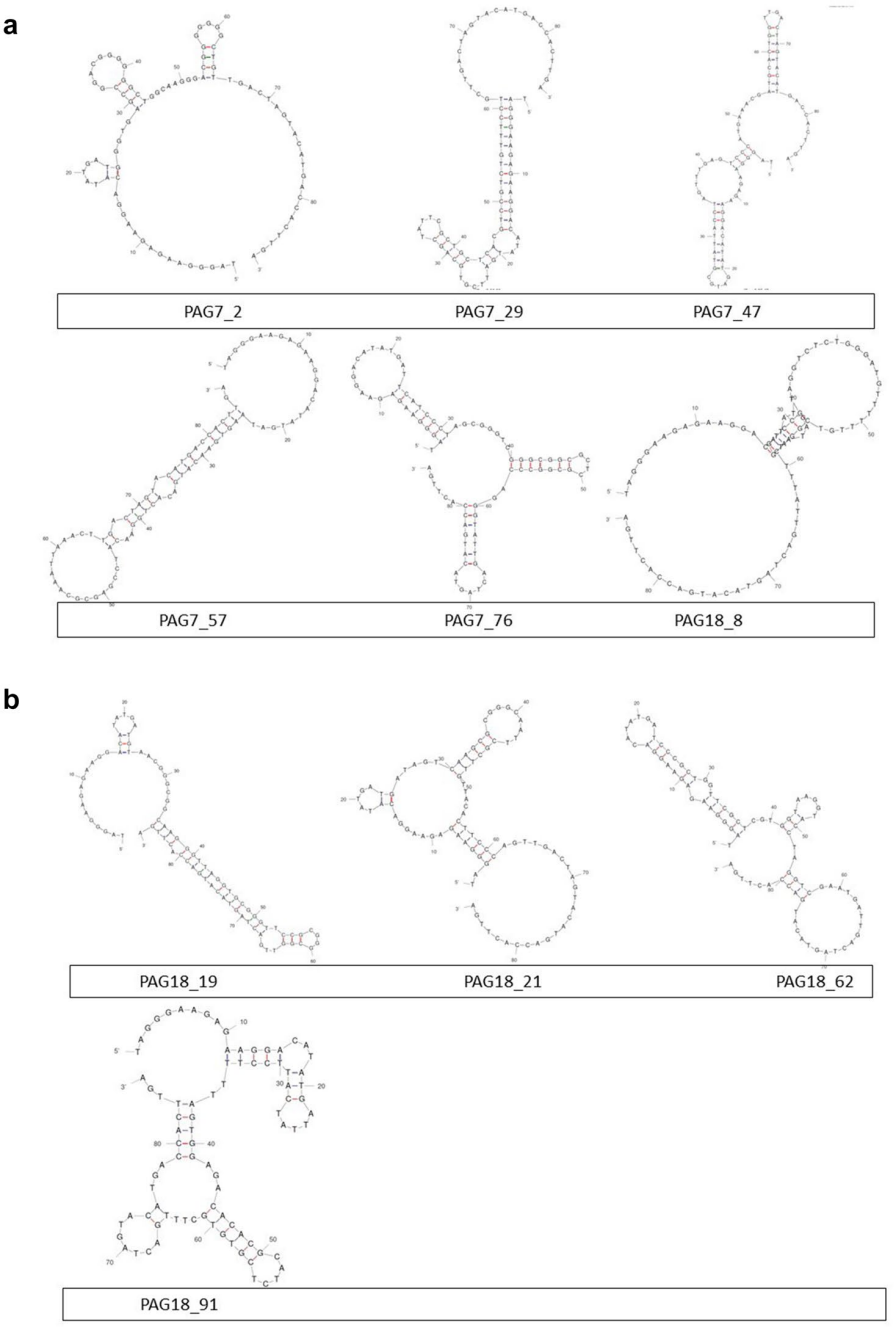
M fold structures of few selected aptamers.

G-Score value of selected aptamers was found to be in the range of 0 to 70 (Table 2, Supplementary File). Further, M-fold structure of each aptamers were also identified (Fig. 4).

**Figure 5:**
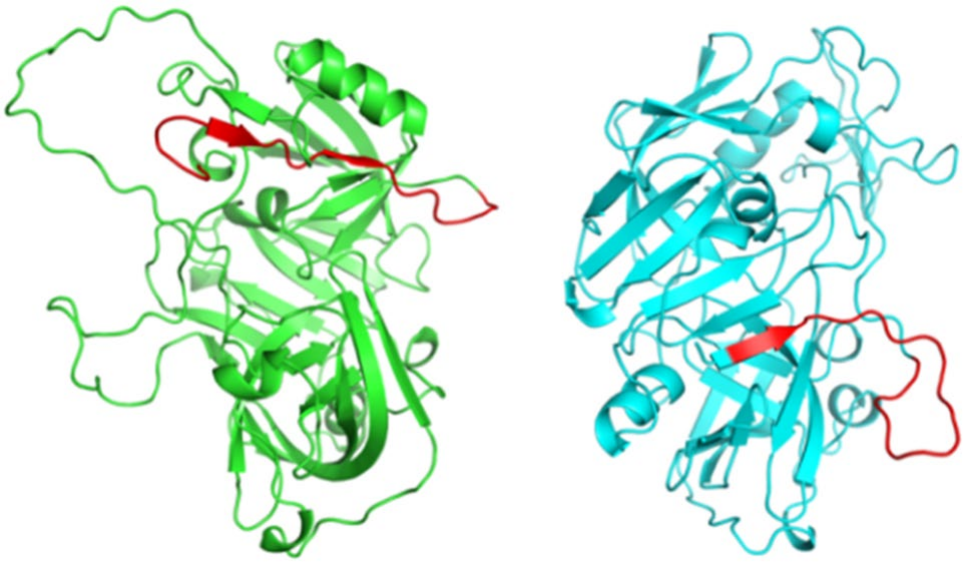
The 3 D structure of PAG 7 and 18 proteins. Highlighted regions represent peptide sequence against which aptamers were selected.

The PAG-7 proteins sequence id A7Y0Q3 and PAG-18 proteins sequence id Q9TTV6 were retrieved from the UniProt database. For modeling the 3-D structure of PAG-7, BLASTp search reveals a template, i.e. the crystal structure of human uropepsin (PDB ID-1FLH) with a sequence identity of 52.02% and query coverage 83%. Whereas in PAG-18, BLASTp search reveals a template, i.e. the crystal structure of human uropepsin (PDB ID- 1FLH) with a sequence identity of 51.71% and query coverage 99%. The 3-D structures were predicted using Modeller v9.24 tool, 50 raw 3-D models developed and the model with the lowest DOPE score model was selected. Further, both structures were loop refined through Galaxy Loop refinement process. The refined models of PAG-7 and PAG-18 were subjected to stereo chemical quality assessment and validation through various protein structure evaluation tools such as PROCHEK, ERRAT, and ProSA while the secondary sequence analysis was done by PSIPRED server. The accuracy of the PAG-7 and PAG-18 models as per PROCHECK was integrated in Ramachandran plot, based on the dihedral (Phi and Psi) angles of each amino acid residues. The PAG-7 model shows 90.6% residues in the most favored regions, 8.1% residues in the additional allowed regions, and 0.6% residues in the generously allowed regions and 0.6% residues in the disallowed region. Whereas in PAG-18 model shows 91.0% residues in the most favored regions, 7.7% residues in the additional allowed regions, 0.6% residues in the generously allowed regions and 0.6% residues in the disallowed region (Fig. 5). In ProSA software, the energy profile analysis gave a Z-score of PAG-7 is − 6.69 and PAG-18 is − 7.34. While the ERRAT score of PAG-7 was 84.50 and for PAG-18 was 80.415.

### Molecular docking

Six aptamers (Random region sequence) were docked against the PAG-7 protein and its peptide sequence while four (Random region sequence) were docked against PAG-18 protein and selected peptide sequence of PAG-18 protein by using Autodock tool (Tables 3 and 4, Supplementary File). The binding affinity score was found more for aptamer–peptide complex as compared to whole proteins.

**Figure 6:**
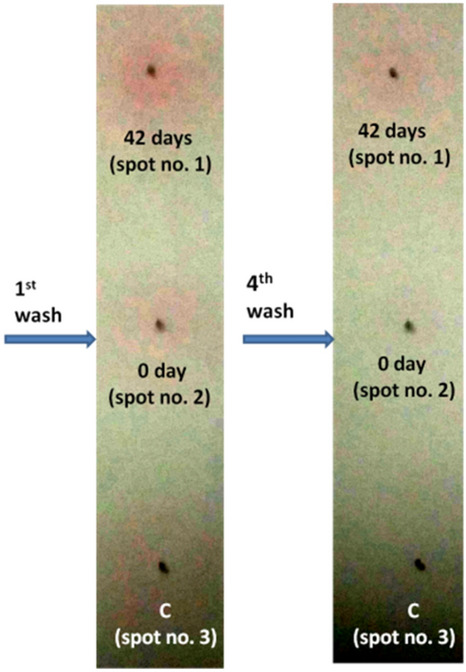
Gold nanoparticle based detection of PAG protein by aptamer PAG7_2.

PAG7_2 recognized the PAG protein in animal whose blood was taken 42nd days of post AI. A clear pink color spot was formed at spot no.1 which stayed up to four washing while such color change was absent in spot no. 2 (0th day animal) and spot no. 3 (control) (Fig. 6).

**Figure 7:**
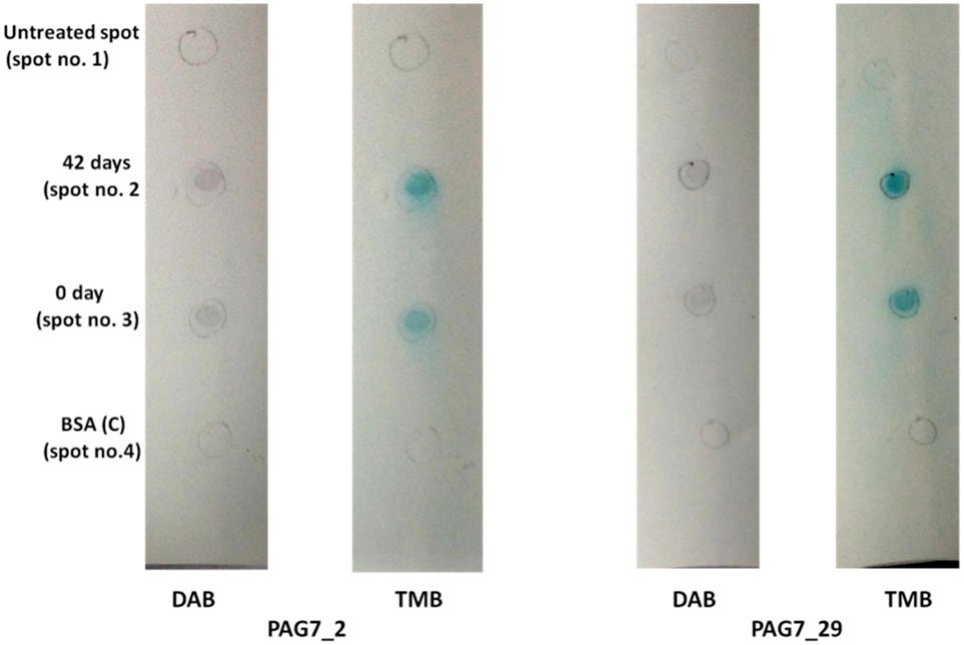
PAG7_2 and 29 interaction with PAG protein of pregnant sample of bovine.

**Figure 8:**
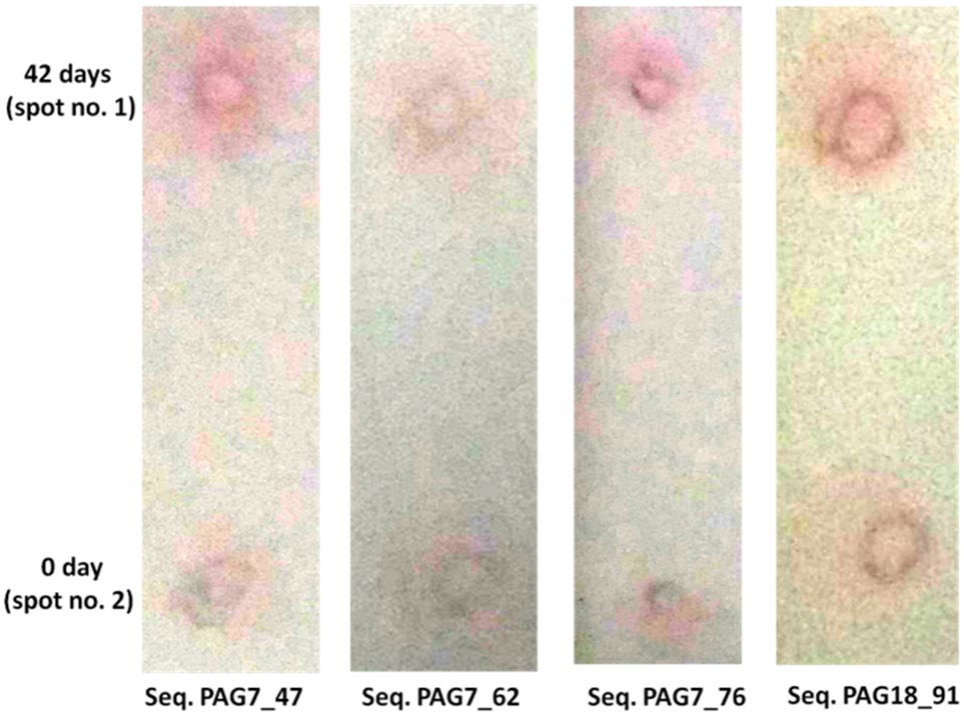
Testing of different aptamers binding affinity for PAG protein in GNP based method.

PAG7_2 and PAG7_29 interacted with PAG protein of pregnant sample of bovine. Both aptamers showed no interaction with membrane or BSA (Fig. 7). The same gold nanoparticle result of PAG7_2 was also observed with PAG7_47, PAG7_62, PAG7_76 and PAG18_91. However spot no. 2 had serum of non pregnant animal which was considered as control (Fig. 8).

**Figure 9:**
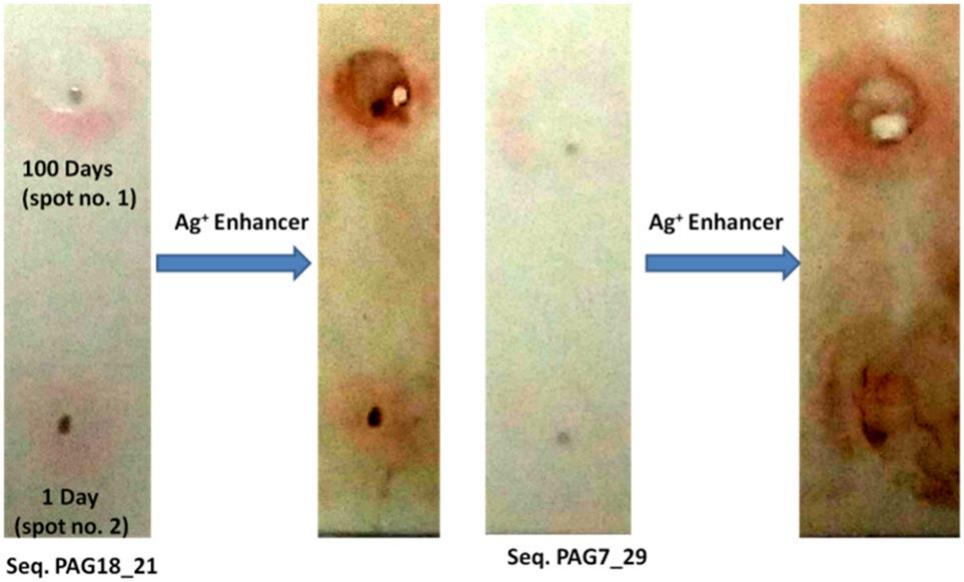
Detection of PAG protein by aptamers in pregnant Water buffalo and postpartum animal.

In ST-GNP method, PAG18_21 and PAG7_29 detected PAG protein of buffalo origin (100th day post AI) and also the PAG protein of an animal that has just given birth to a calf (1 day post calving). The intensity of PAG protein becomes more prominent when silver enhancer was used (Fig. 9).

**Figure 10:**
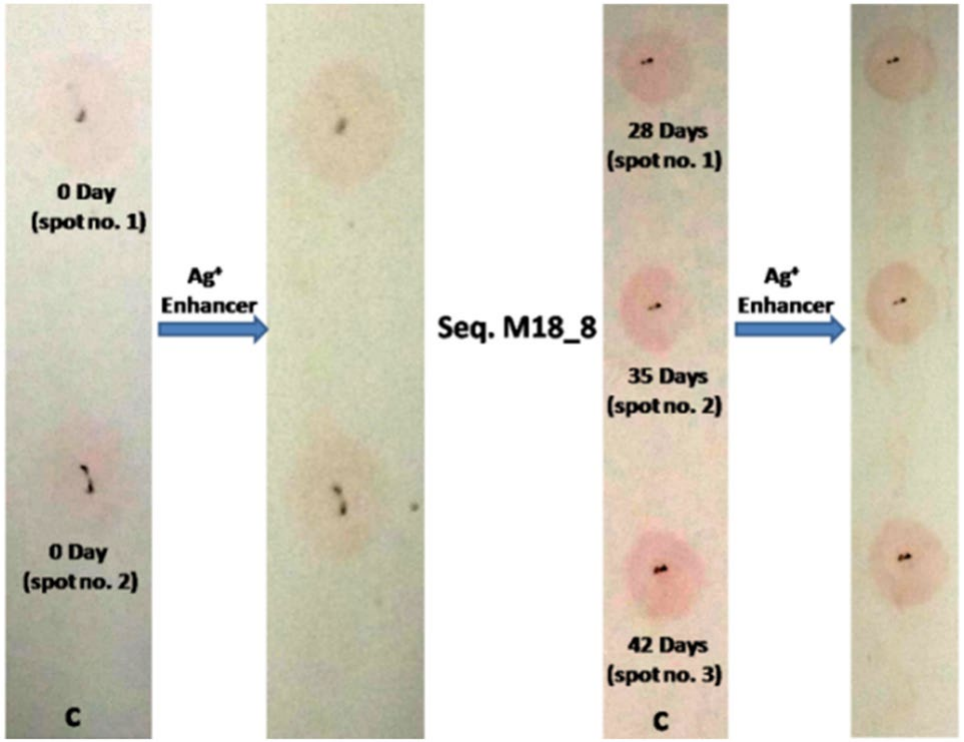
Detection of PAG protein by aptamers at 0 day and at different days after AI.

In another ST- GNP approach, aptamer PAG18_8 detected PAG protein in 28th, 35th and 42nd days serum sample of animal’s after their AI. The color intensity was found more in 35th and 42nd days samples as compared to 28th days. Further minimal color was observed in 0th day animals (Fig. 10).

**Figure 11:**
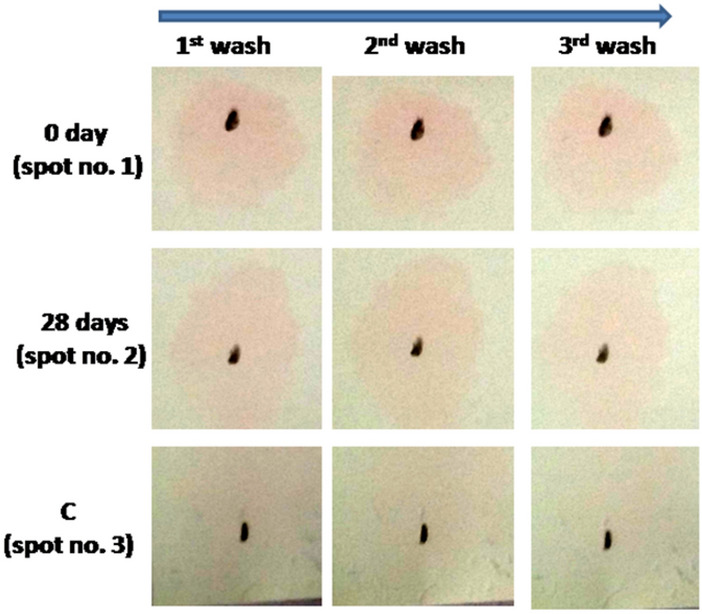
Time dependent interaction study of aptamer with PAG protein.

In another experiment, PAG18_8 detected PAG protein of 28th day sample in 20 min incubation. However no color was observed in control after third wash (Fig. 11).

**Figure 12:**
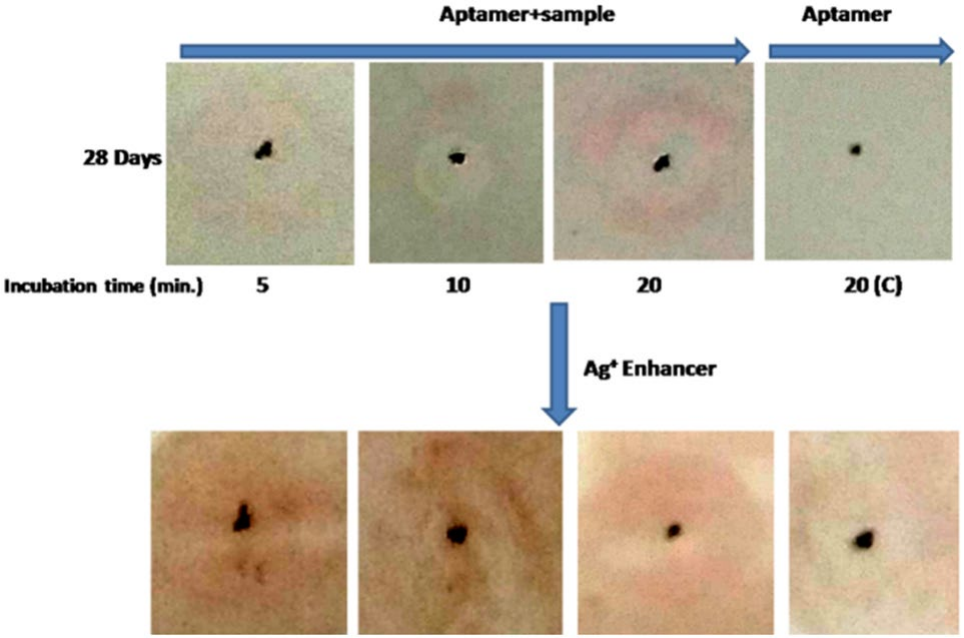
Use of nylon membrane for detection of PAG protein by aptamer.

Aptamer PAG7_47 showed minimum interaction to component of 0th day animal serum sample. However its interaction with PAG protein was observed as intensity of GNP was found more on spot no. 1 as compared to 2. This is due to GNP conjugated sequence (complementary of random region of PAG7_47) had more access to aptamer PAG7_47 immobilized on spot no.1 as compared to spot no. 2 on membrane. Further, GNP conjugated sequence had little interaction with untreated membrane surface (Fig. 12).

**Figure 13:**
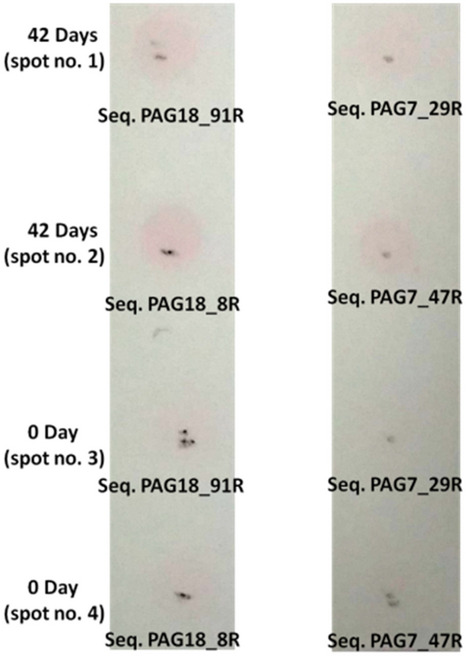
Random region of aptamer’s interacting with PAG protein.

In competitive color experiment, all the sequences showed little interaction with serum of 0th day animal. The interaction with PAG protein (serum of animal after passing 42 days of AI) was found prominent with Seq. named PAG18_8R, PAG18_91R and PAG7_47R (Fig. 13).

**Figure 14:**
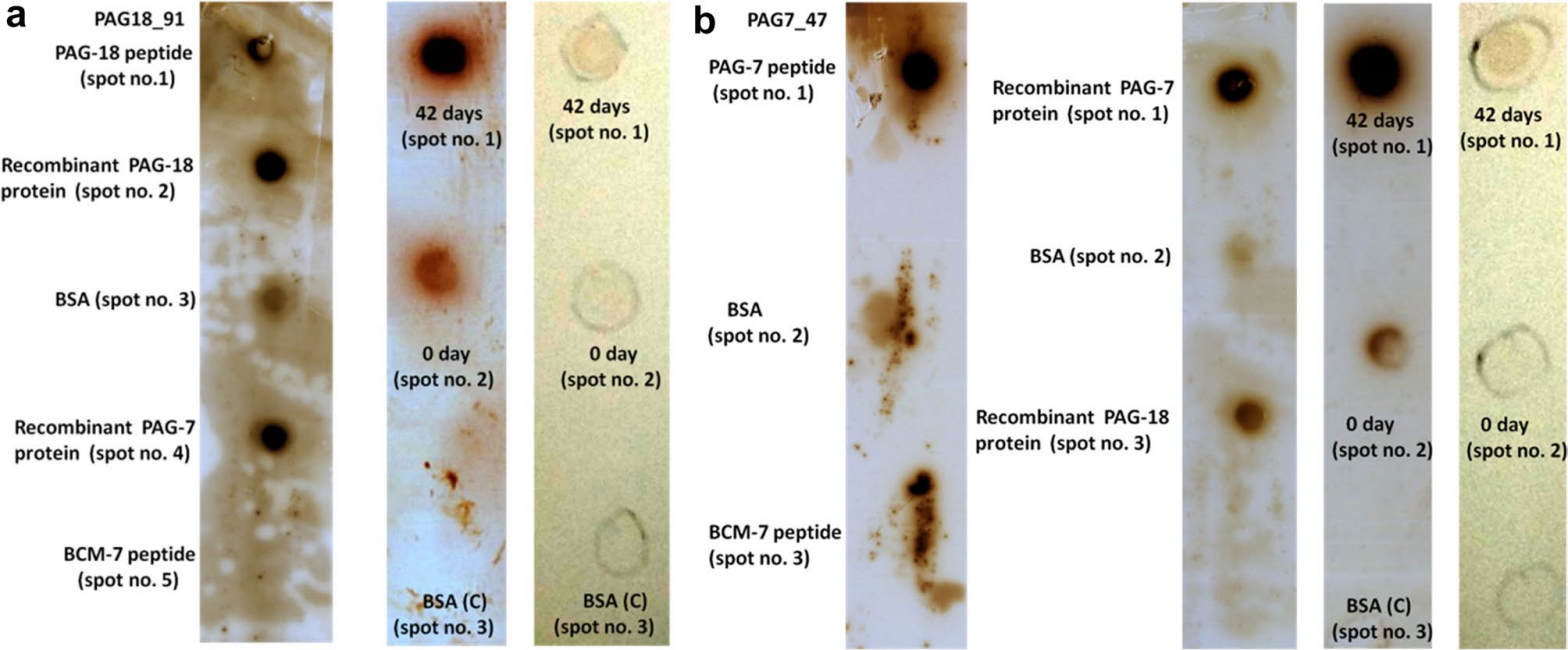
Detection of PAG-18 (**a**) and 7 (**b**) proteins by aptamers in ECL and DAB coloring agents.

In ECL and DAB experiment, PAG18_91 (Fig. 14a) and PAG7_47 aptamers (Fig. 14b) have given intense color with pregnant animal’s serum as compared to non pregnant ones. The respective aptamers also showed their interaction with PAG peptide and recombinant PAG proteins.

## Conclusion

Antibodies were selected against the said peptides of the PAG proteins in previous unpublished work from same lab. The antibodies showed interaction with proteins. It is hypothesized that if a large molecule can interact with peptide, a small molecule like aptamer should also interact with the selected peptide. The molecular docking studies show that aptamers are more specific to peptide sequence against which they are selected as compared to whole protein. On that basis, aptamers were selected against peptide sequence of PAG-7 and 18 proteins in current study. Liu et al.^15,16^ has selected aptamers against PAG 4 and 5 proteins through SELEX method. They have developed ELISA for detection of PAG proteins. In current selection method aptamers were selected against specific segment of PAG protein instead of whole protein in magnetic bead based approach through Non-SELEX method. The method uses color based approach for selection of aptamer against the target. It further, gives the information regarding aptamers interaction with peptide with no color change in control tube. However selection of few aptamers for biosensor development after sequencing is a tedious job. An attempt has been made to select aptamers on the basis of complex M fold structure. In other methods of single step SELEX either florescence approach or physical process was PCR amplification was carried out of segmented affinity column that contains aptamer-target complex. These methods are reliable but tedious to perform. Fluorescence approach is good but at the same time it gets quenched very quickly; further breaking the cover slip and using it for amplification can be cumbersome. In monoLEX method also aptamers can interact with resin of column non-specifically and give amplification. Breaking column and using it for amplification further adds additional work.

The selected aptamers tested on serum of 0th day and 42nd day animals post AI. All aptamers showed interaction with PAG proteins. The random region of aptamers was also able to interact to PAG protein of pregnant animal. Although we also observed little interaction with serum of 0 day animal, however the intensity of interaction was very low. This was due to variability in AI that was performed on different time duration on given animals after their parturition as most of the PAGs are removed from the animals only after 80–100 days of calving^1^. Results also showed aptamers are capable to interact with PAG proteins at much lower days (such as day 28) after AI of an animal. The selected peptide sequence for aptamer selection is also present in others PAGs, as aptamers selected from PAG-18 peptide showed interaction with PAG-7 protein. This is not a demerit of aptamers. Instead, aptamers interaction with multiple forms of PAG proteins increases the intensity of interaction with PAG proteins of serum sample of pregnant animal. Further, it has been found that PAG18_91, PAG18_8 and PAG7_47 showed least interaction with components of serum of non pregnant sample and can be considered as good candidate for development of pregnancy detection kit for animals. Also, dot blot method is an easy and inexpensive way of detecting pregnancy in animals.

## Supplementary Information

Supplementary Information 1.

Supplementary Information 2.
