# Supplementary material for: Aptamers based sensing of pregnancy associated glycoproteins (PAG) of bovine for early pregnancy detection
Source: Sci Rep. 2021 Dec 1;11:23193. doi: 10.1038/s41598-021-02551-1 (PMC8636505; doi:10.1038/s41598-021-02551-1)
Supplement: Supplementary file 1 — Supplementary Information 1. [file 41598_2021_2551_MOESM1_ESM.docx]

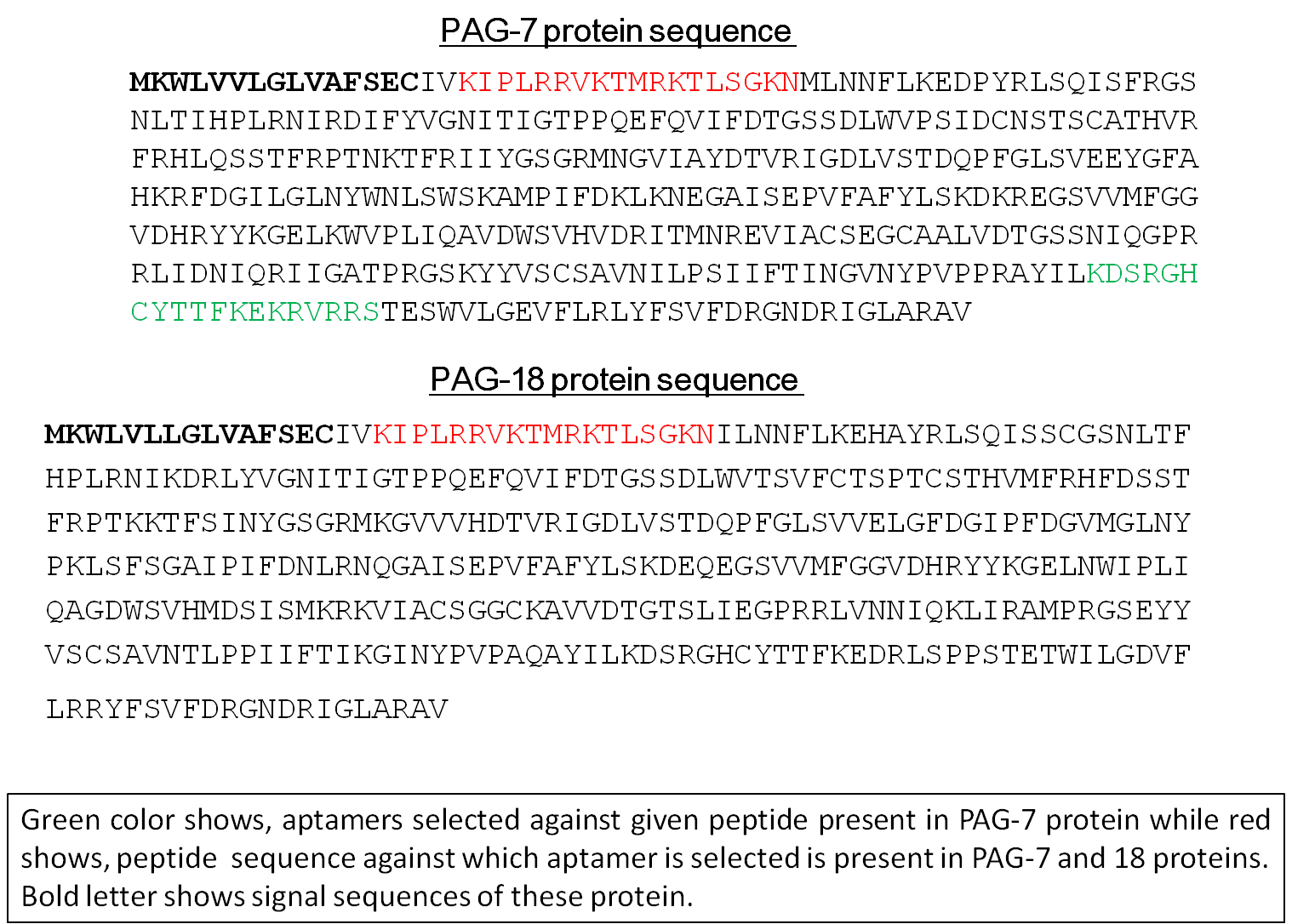


**Peptide sequences selected from PAG proteins for aptamer selection**

**Table 2: G- Score of selected aptamers**

| **Aptamer name** | **G-score** |
| --- | --- |
| PAG**7_2** | **68** |
| PAG**7_29** | **0** |
| PAG**7_47** | **0** |
| PAG**7_76** | **34** |
| PAG**18_91** | **0** |
| PAG**7_57** | **0** |
| PAG**18_19** | **70** |
| PAG**18_21** | **0** |
| PAG**18_62** | **30** |
| PAG**18_8** | **30** |

**Table 3 : PAG protein and aptamers docking result**

| **Protein Name** | **Aptamer Sequence (Random region)** | **Binding Affinity Score** |
| --- | --- | --- |
| PAG-7 | GGGTGAGCCGGACGGGGGGCTGGCAAGGGACGGGGGGGCTG (PAG7_2) | -11.2 |
|  | TCGTGCAGCTATTCGCTGCTCACGTCCGTCTGTTTCCTGC (PAG7_29) | -10.7 |
|  | GCGTATTACCTAGTTTGAGTCCCATGAAACGATGCACTGG (PAG7_47) | -11.3 |
|  | AAGTGAACATGACACTGGAACATCCGAGCGCAAATTAAAC (PAG7_57) | -10.5 |
|  | TCATCCCTAGCGGGTCGGGCGGCGCTCGCGGCCCAGGGTA (PAG7_76) | -11.9 |
| PAG-18 | ATGGTCCGTAGGTCTCTGGGATGTTTTTTGTCGGACT (PAG18_8) | -10.6 |
|  | GTAACGGGCGGCAAGGGTTAGGTGCGGGTTCCGCGGGCGG (PAG18_19) | -10.3 |
|  | GATAGTCAAGCGCGGGCAATTCGCTTGTTACACTTCCCAG(PAG18_21) | -10.8 |
|  | TATCATTCCTTTTAGTGGAGACACACGCATCTCGTGTGCT (PAG18_91) | -10.9 |
|  | CCCGCTGGTTCGCTCGTGGTAAGGTACCTAGGTCGAATGA (PAG18_62) | -10.4 |

**Table 4 : PAG peptide and aptamers docking result**

| **Protein Name** | **Aptamer Sequence (Random region)** | **Binding Affinity Score** |
| --- | --- | --- |
| PAG-7 peptide | GGGTGAGCCGGACGGGGGGCTGGCAAGGGACGGGGGGGCTG (PAG7_2) | -14.0 |
|  | TCGTGCAGCTATTCGCTGCTCACGTCCGTCTGTTTCCTGC (PAG7_29) | -14.6 |
|  | GCGTATTACCTAGTTTGAGTCCCATGAAACGATGCACTGG (PAG7_47) | -14.2 |
|  | AAGTGAACATGACACTGGAACATCCGAGCGCAAATTAAAC (PAG7_57) | -14.5 |
|  | TCATCCCTAGCGGGTCGGGCGGCGCTCGCGGCCCAGGGTA (PAG7_76) | -12.7 |
| PAG-18 peptide | ATGGTCCGTAGGTCTCTGGGATGTTTTTTGTCGGACT (PAG18_8) | -11.4 |
|  | GTAACGGGCGGCAAGGGTTAGGTGCGGGTTCCGCGGGCGG (PAG18_19) | -12.0 |
|  | GATAGTCAAGCGCGGGCAATTCGCTTGTTACACTTCCCAG (PAG18_21) | -12.3 |
|  | TATCATTCCTTTTAGTGGAGACACACGCATCTCGTGTGCT (PAG18_91) | -12.0 |
|  | CCCGCTGGTTCGCTCGTGGTAAGGTACCTAGGTCGAATGA (PAG18_62) | -12.5 |

**Fig. 2 Magnetic bead based colorimetric method for aptamer selection against PAG-7 protein**


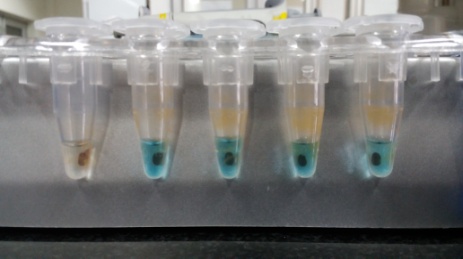

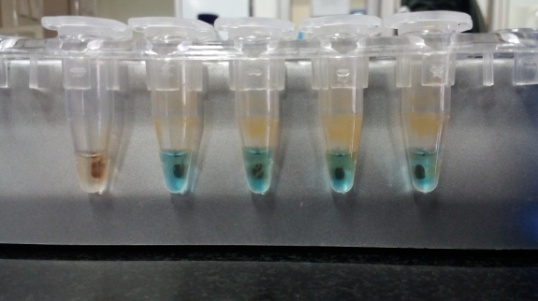

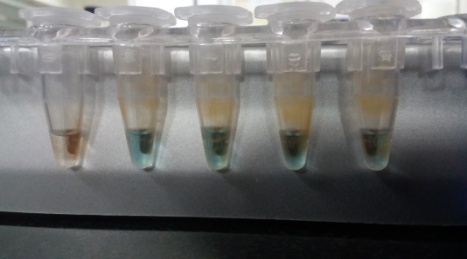

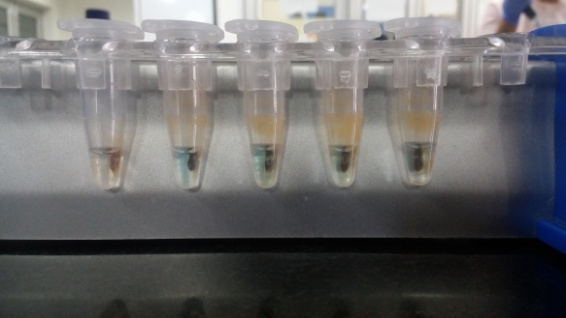

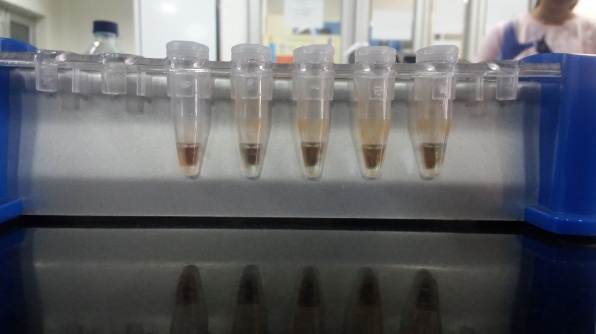

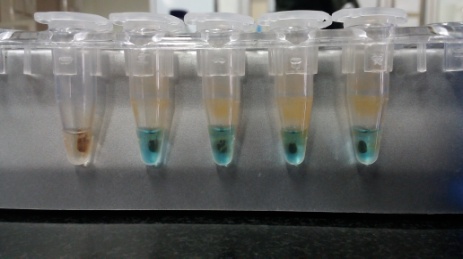

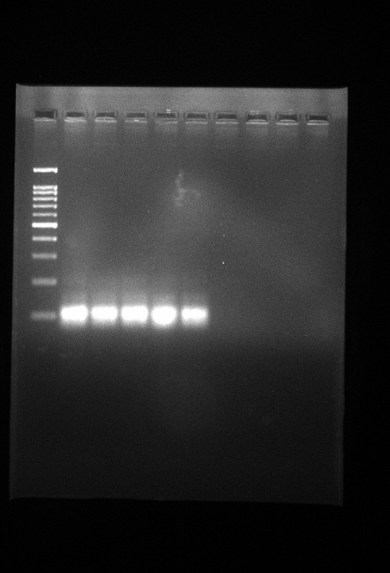

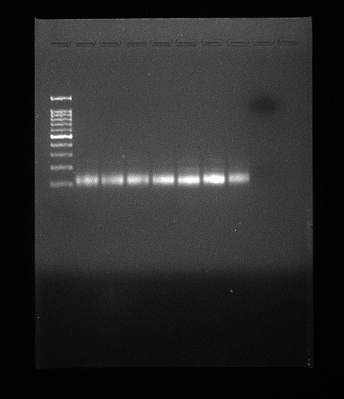

**Fig. 3 Agarose gel run of amplified aptamers, selected against PAG proteins**


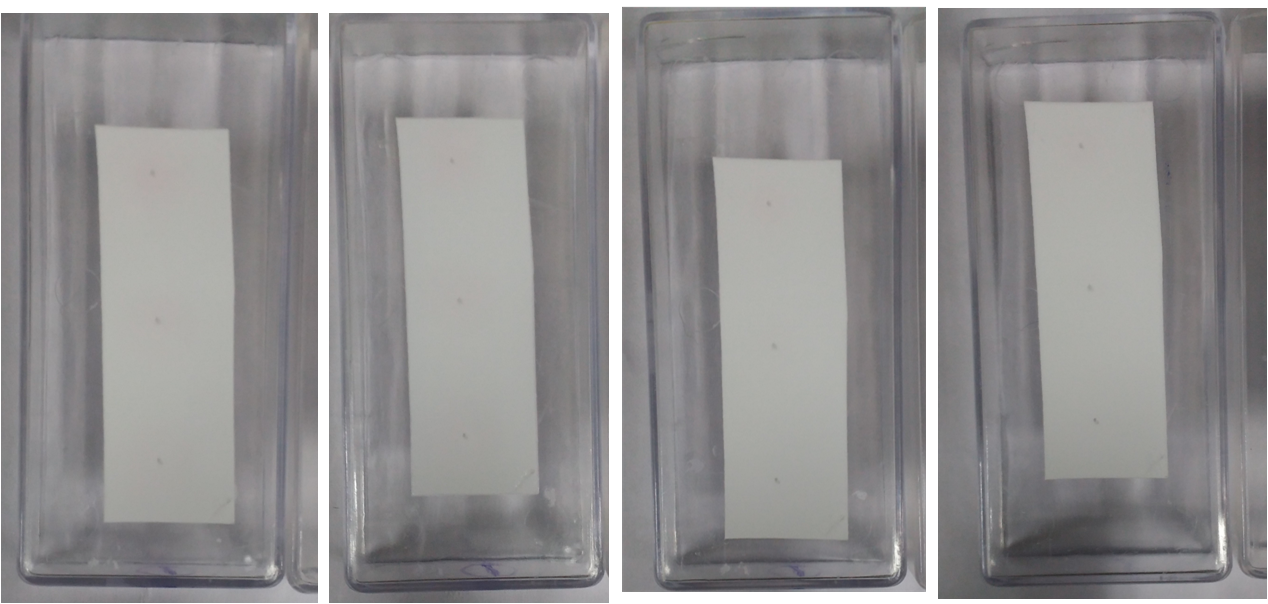


**42 days (PAG 7_2)**

**0 day (PAG 7_2)**

**C**

**4^th^ wash**

**3^rd^ wash**

**2^nd^ wash**

**1^st^ wash**

**Fig. 6 Gold nanoparticle based detection of PAG protein by aptamer PAG7_2**


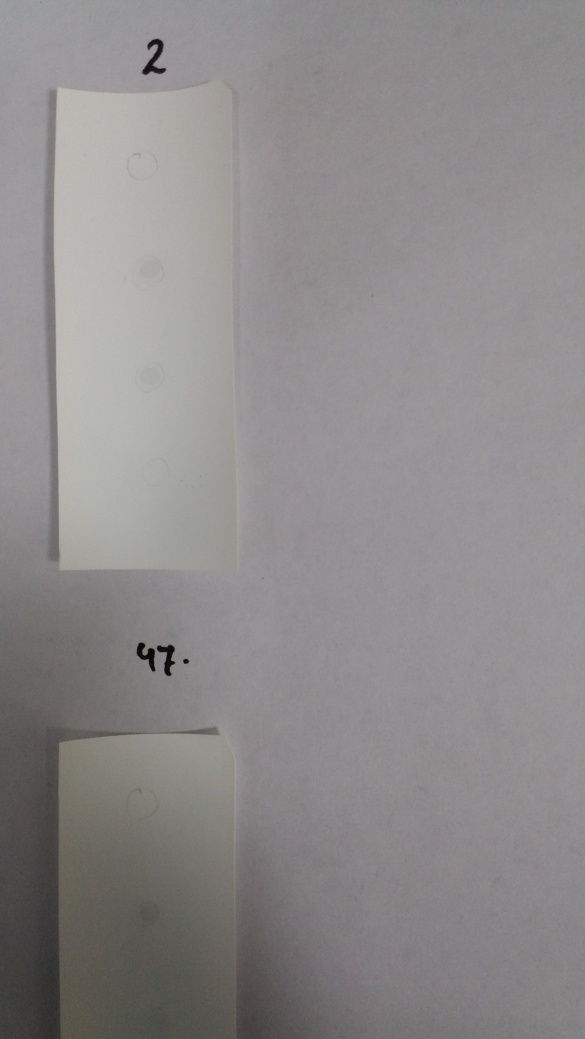

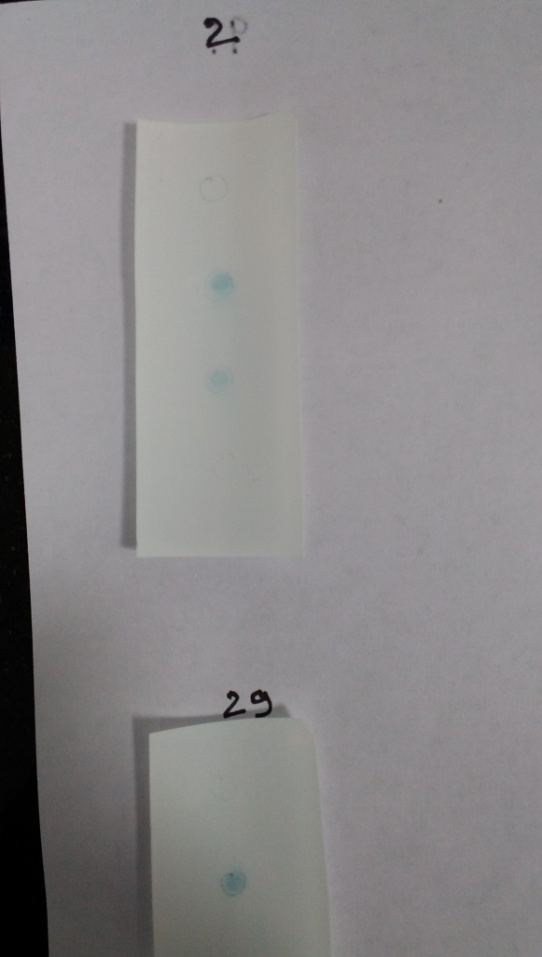

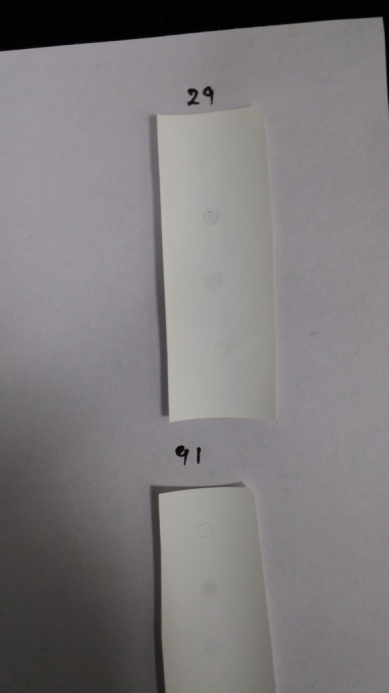

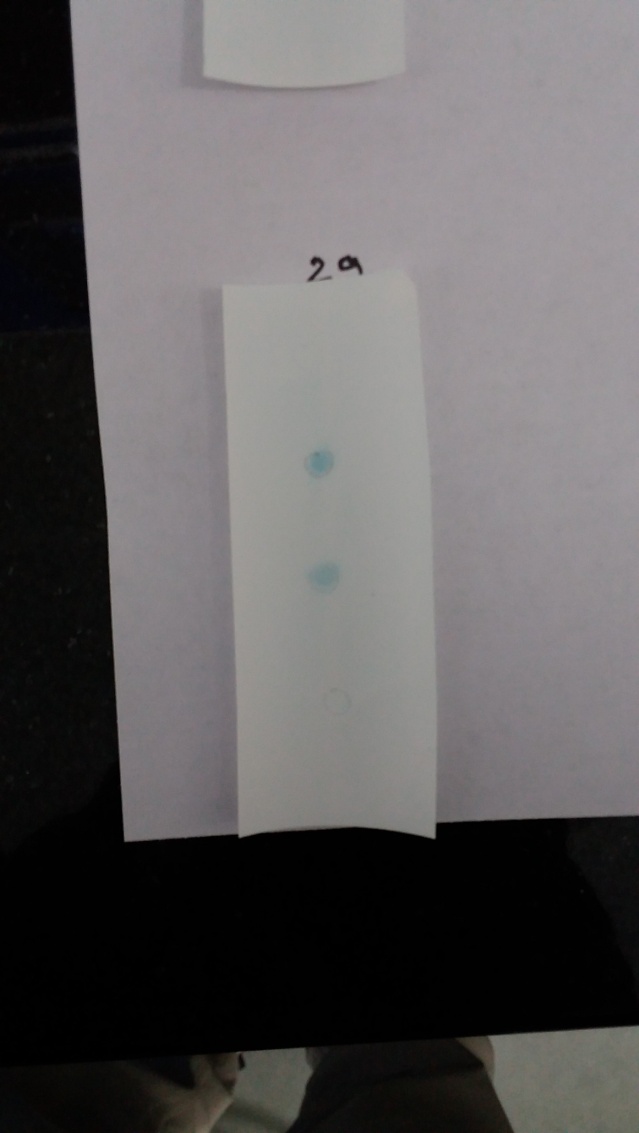


**Untreated spot**

**42 days**

**0 day**

**BSA**

**Fig. 7a PAG7_2 & 29 interaction with PAG protein of pregnant sample of bovine**


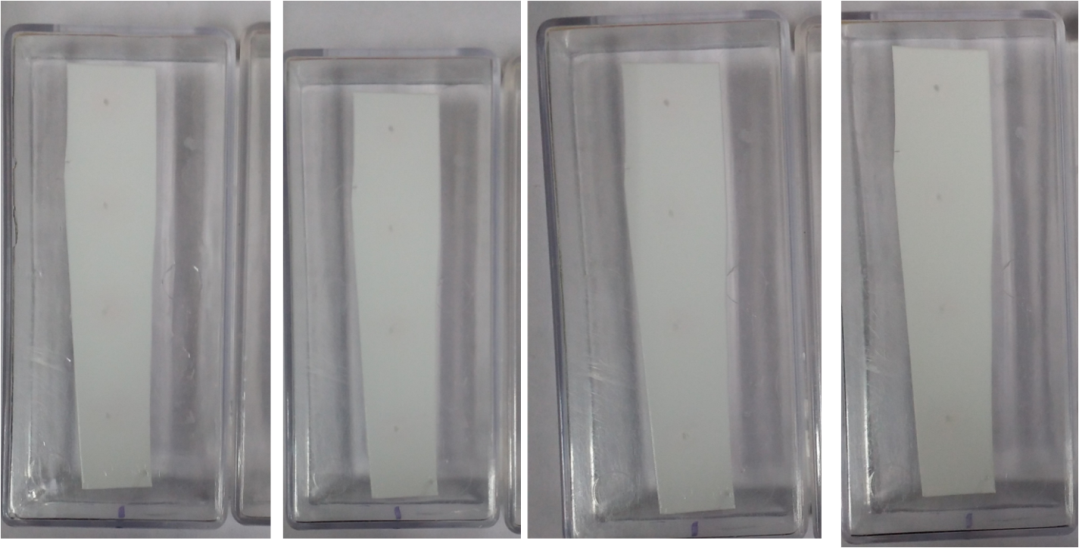


**42 days (PAG7_29)**

**0 day (PAG7_29)**

**1^st^ wash**

**4^th^ wash**

**2^nd^ wash**

**3^rd^ wash**

**Fig. 7b PAG7_29 detection of PAG protein in GNP based assay**

**3^th^ wash**

**2^nd^ wash**

**1^st^ wash**

**42 days (PAG7_47)**

**0 day (PAG7_47)**


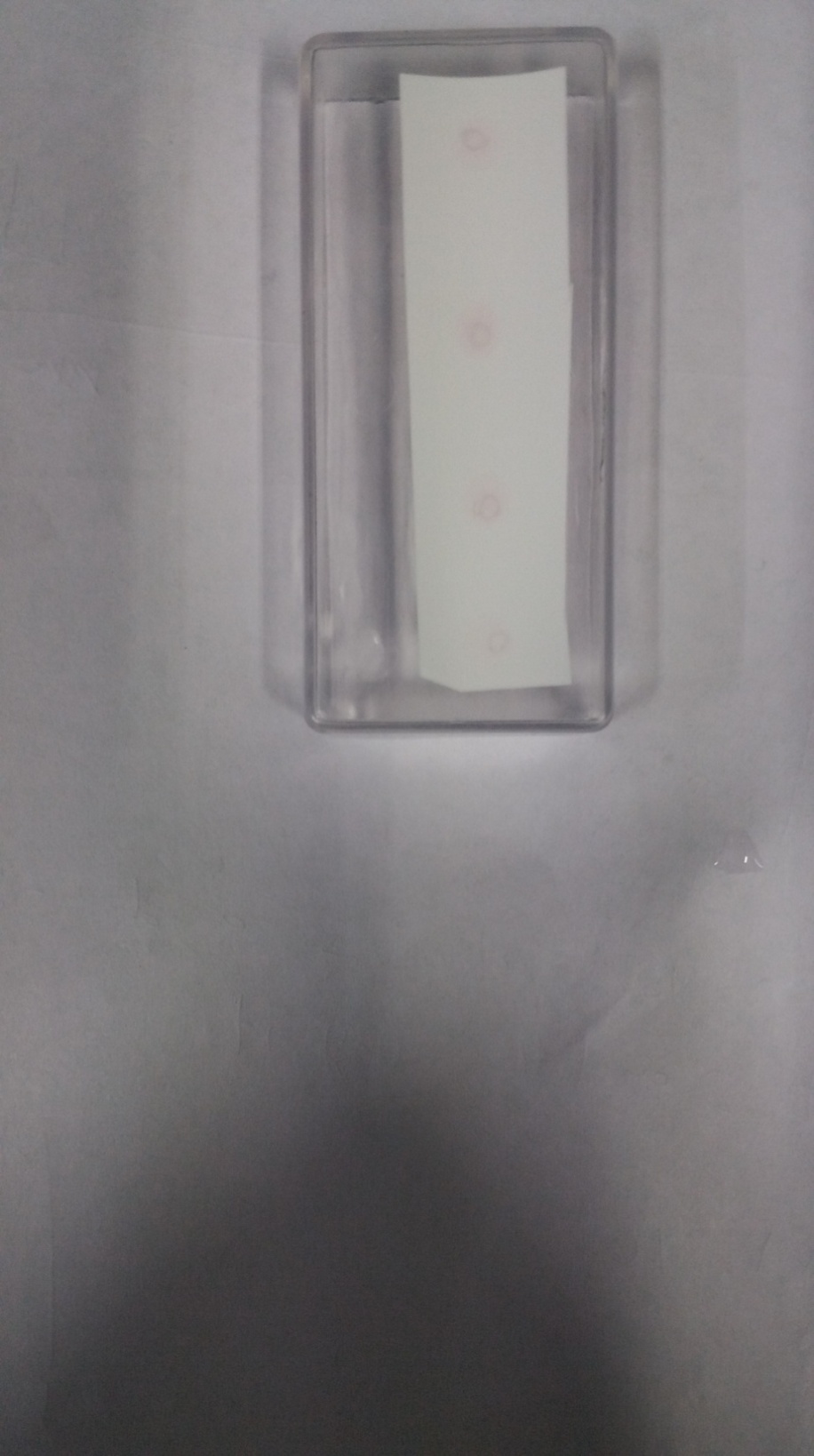


**42 days (PAG7_62)**

**0 day (PAG7_62)**

**42 days (PAG18_91)**

**0 day (PAG18_91)**

**42 days (PAG7_76)**

**0 day (PAG7_76)**

**Fig. 8 Testing of different aptamers binding affinity for PAG protein in GNP based method**


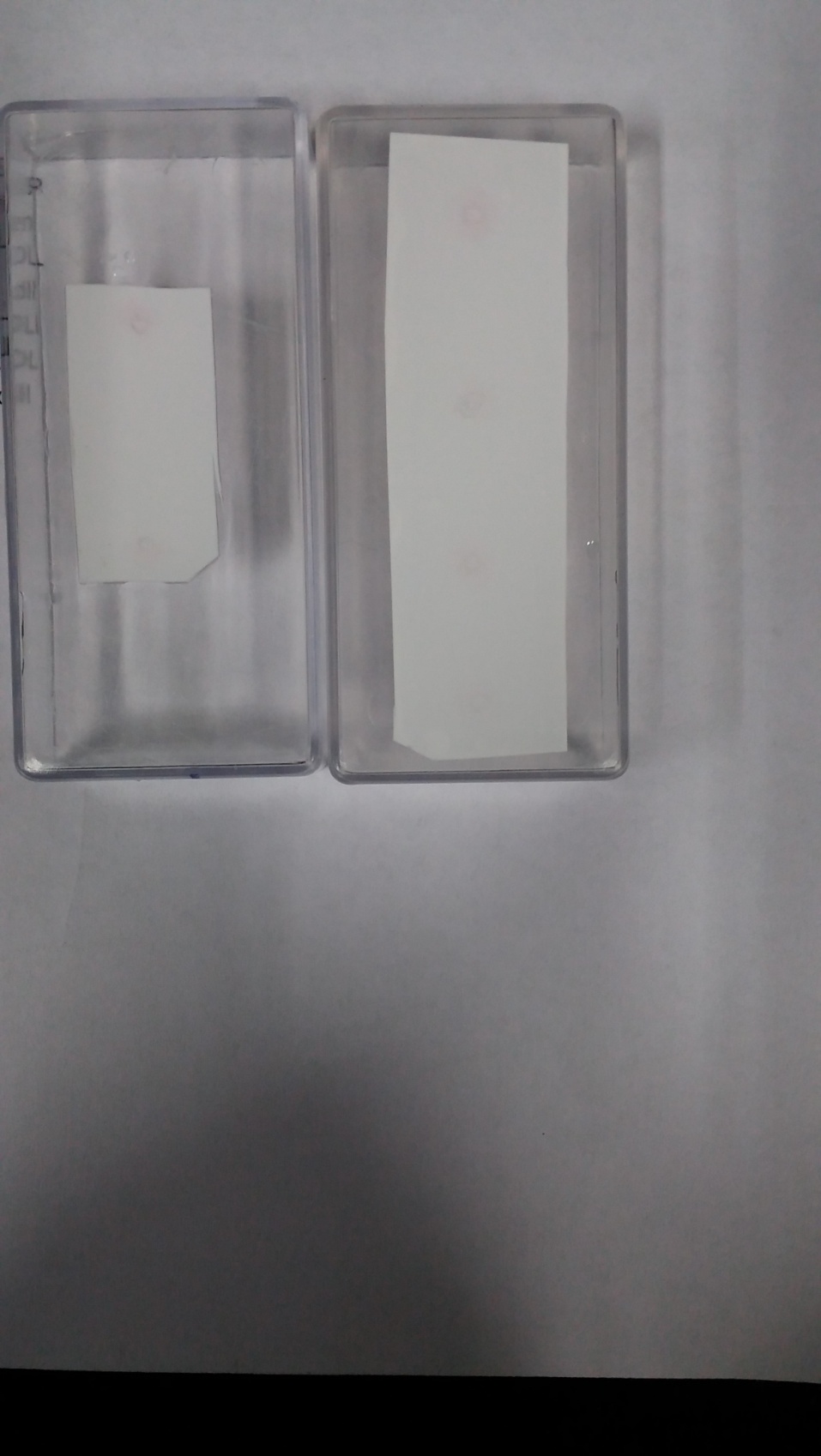


**100 days ( PAG 18_21)**

**1 day (PAG 18_21)**

**100 days ( PAG 7_29)**

**1 day (PAG 7_29)**

**Ag^+^ enhancement**

**(PAG 7_29)**

**Ag^+^ enhancement ( PAG 18_21)**


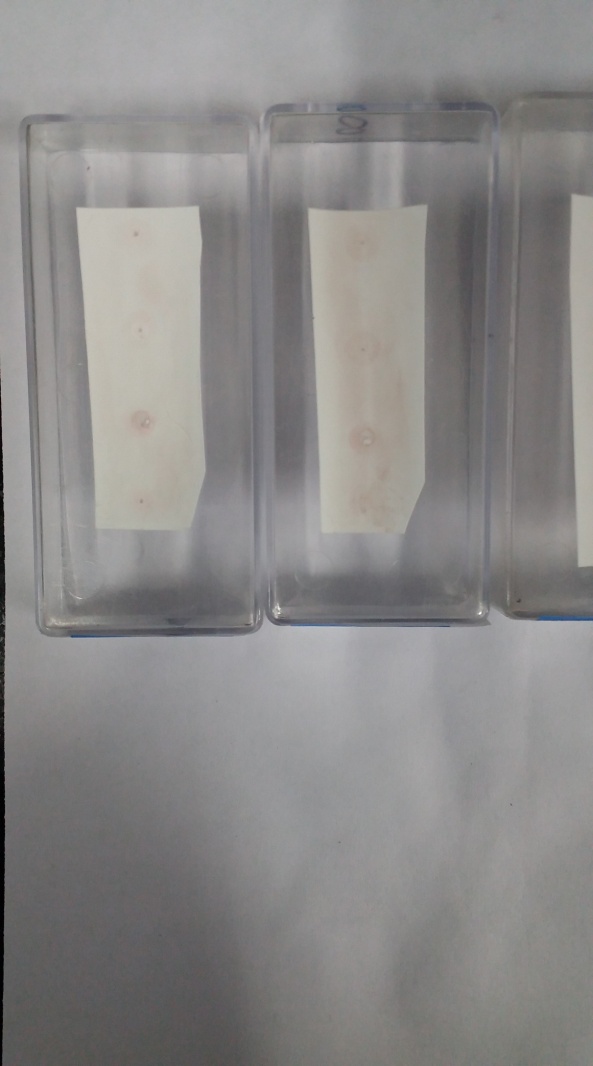

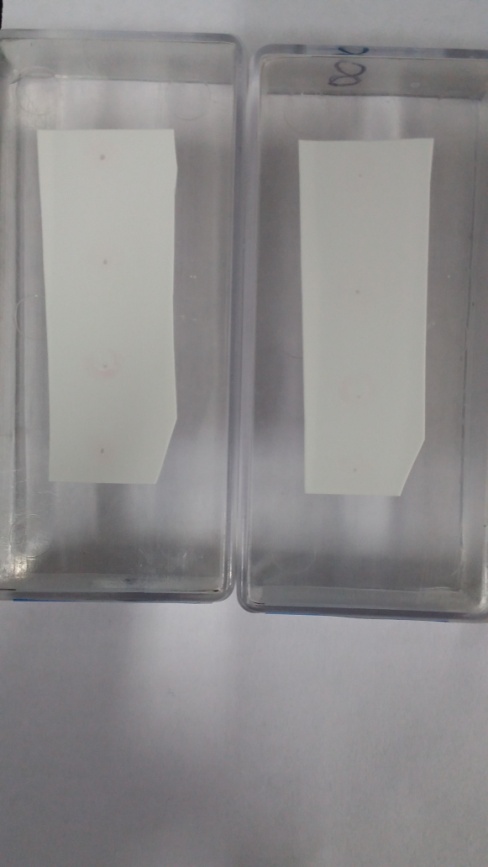


**Fig. 9a Detection of PAG protein by aptamers in pregnant Water buffalo and postpartum animal**


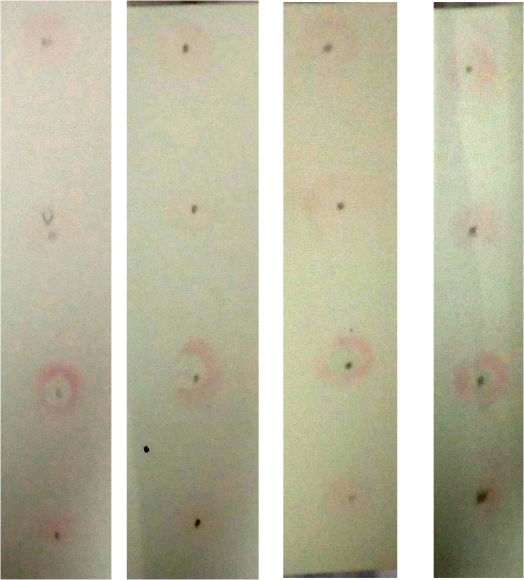


**100 days ( PAG 18_21)**

**1 day (PAG 18_21)**

**100 days ( PAG 7_29)**

**1 day (PAG 7_29)**

**Fig. 9b Other pregnant samples of water buffalo that were used to see PAG interaction with PAG18_21 and PAG7_29**


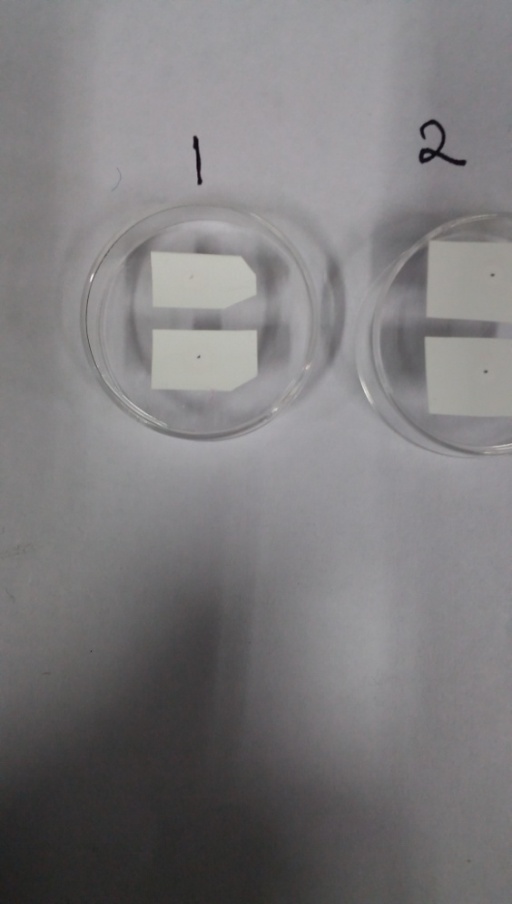

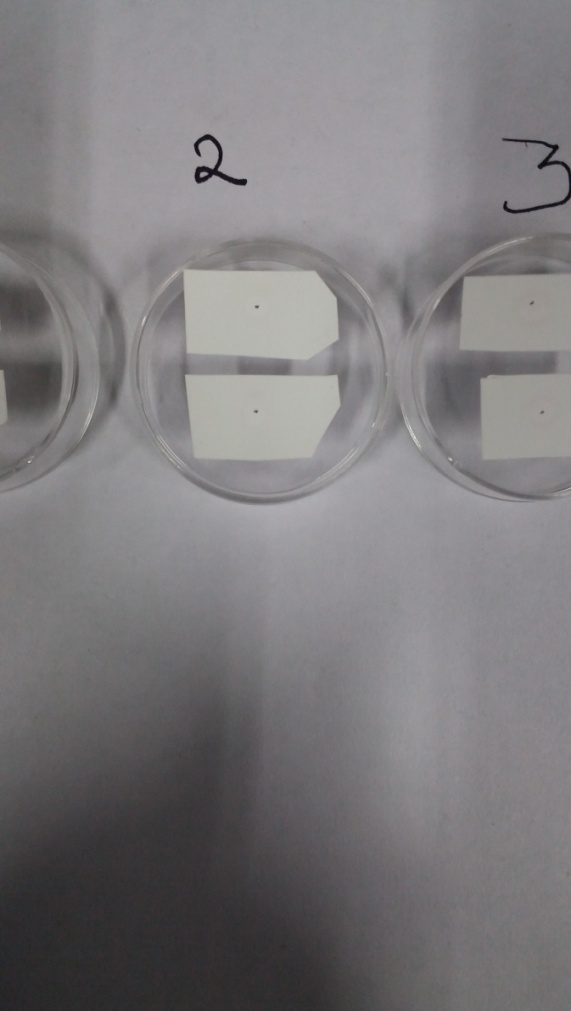


**Incubation time 5 10 20 20(c)**

**1 2 3 4**


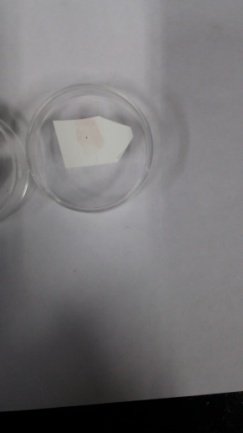

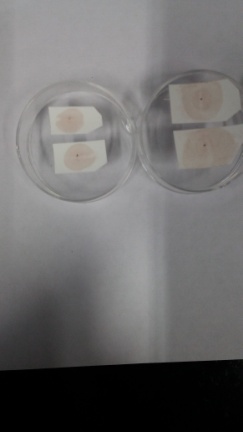


**Ag^+^ enhancement**


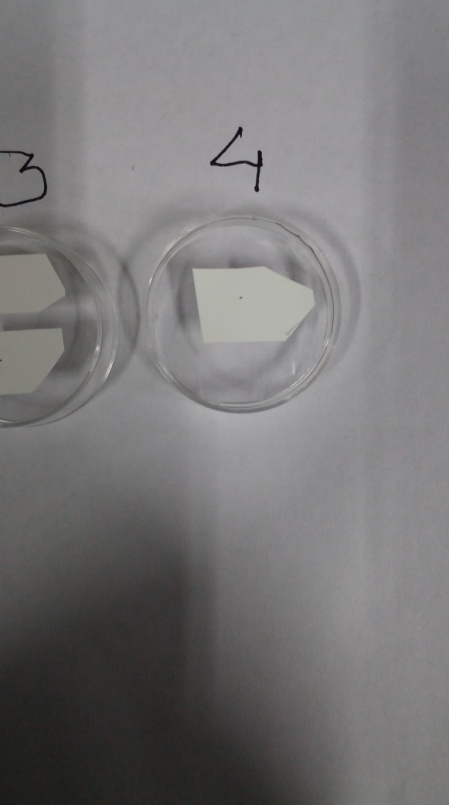

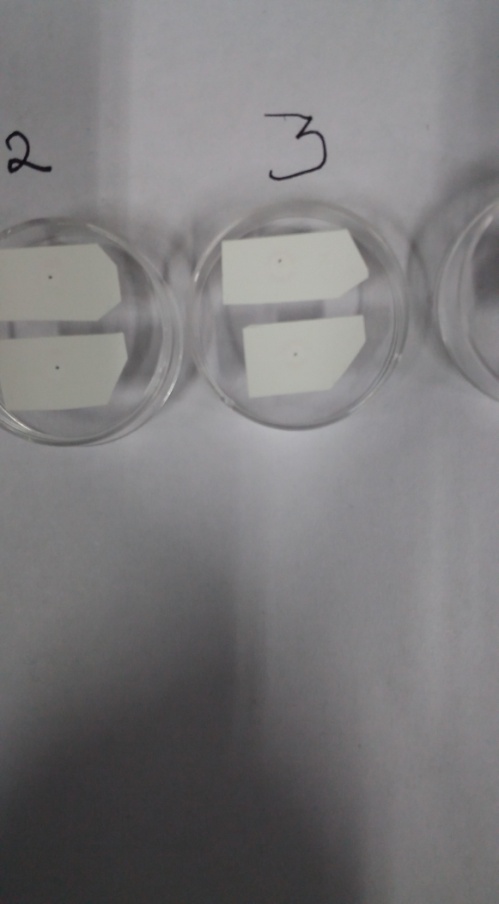

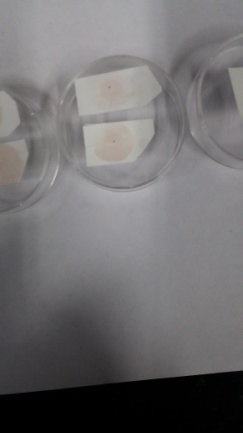

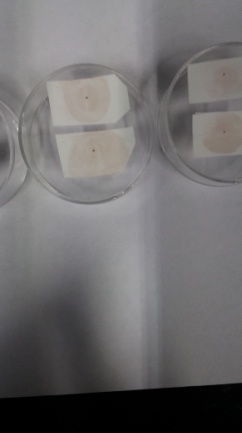


**Fig. 11 Time dependent interaction study of aptamer with PAG protein**


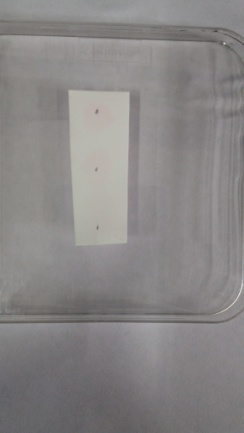


**0 day**

**28 days**

**C**

**4^st^ wash**

**3^rd^ wash**

**2^nd^ wash**

**1^st^ wash**

**Fig. 12 Use of nylon membrane for detection of PAG protein by aptamer**


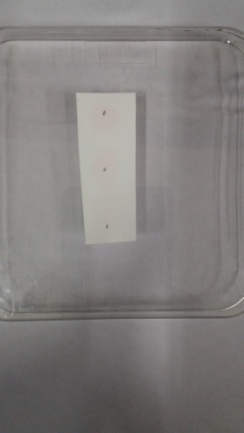

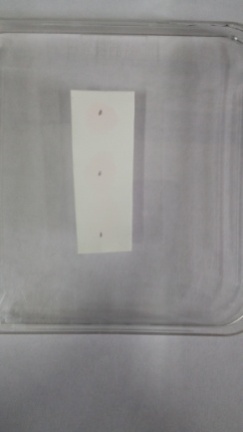

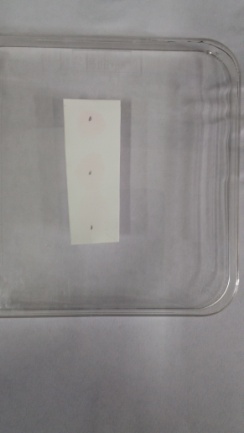


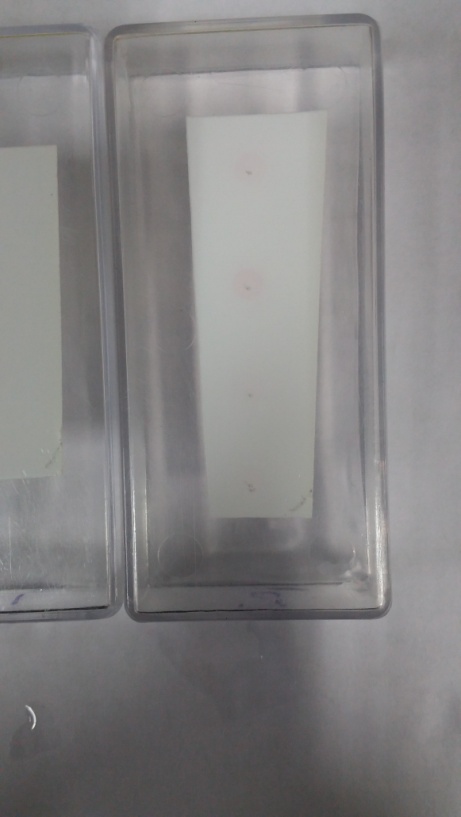

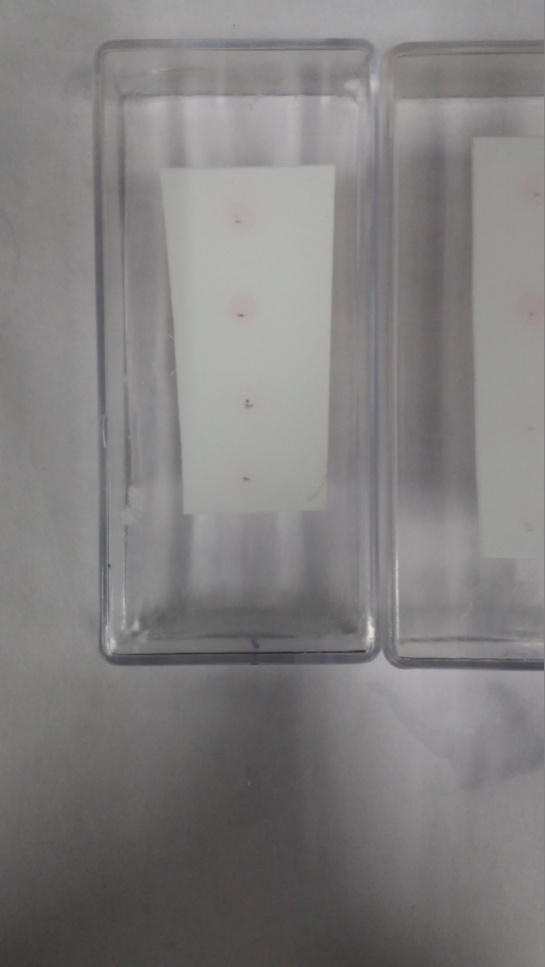


**42 days (PAG 18_19R)**

**42 days ( PAG 18_8R)**

**0 day (PAG 18_91R)**

**0 day ( PAG 18_8R)**

**42 days (PAG 7_29R)**

**42 days (PAG 7_47R)**

**0 day (PAG 7_29R)**

**0 day ( PAG 7_47R)**

**Fig. 13 Random region of aptamer’s interacting with PAG protein**

**Fig. 15**


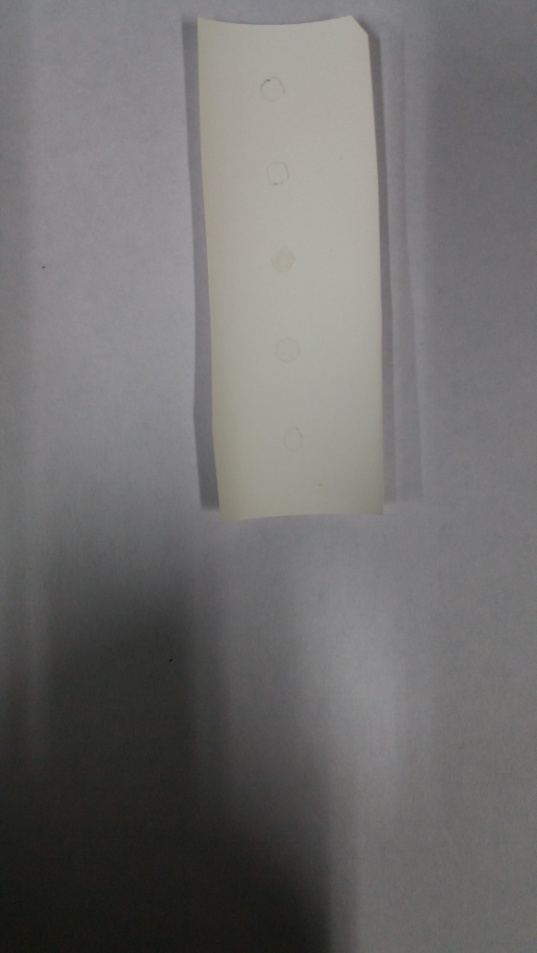

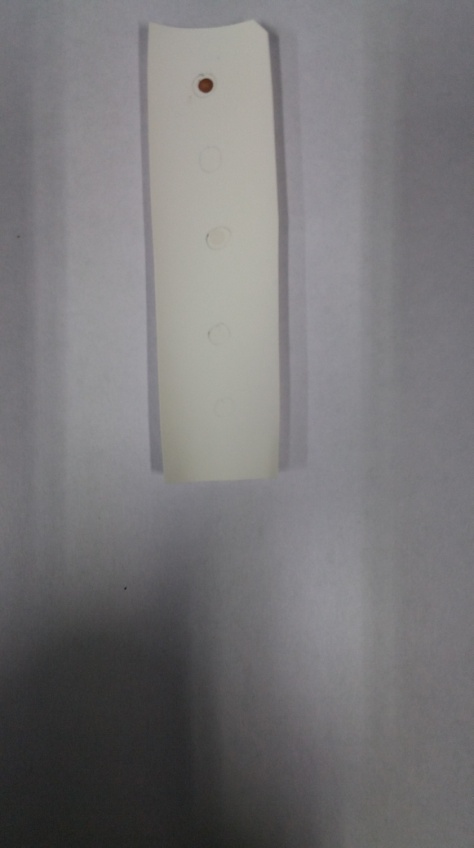

**PAG-7 peptide (PAG 7_47)**

**BSA (C)**

**42 days (PAG7_47)**

**0 day (PAG7_47)**

**BSA**

**42 days (PAG 18_91)**

**0 day (PAG 18_91)**

**BSA (C)**

**Detection of PAG protein by aptamers in presence of DAB chemical**


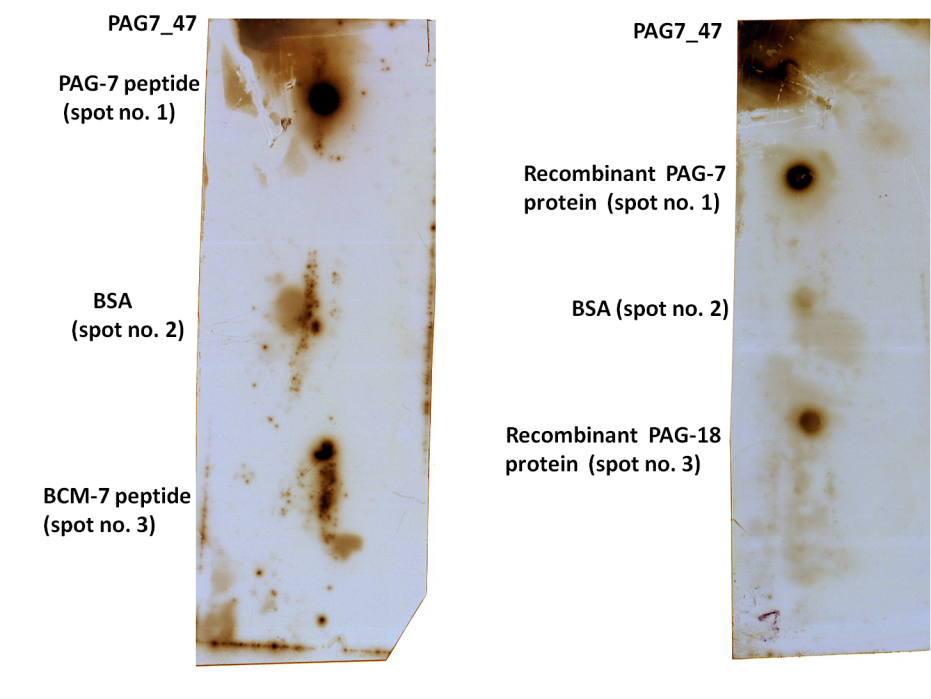

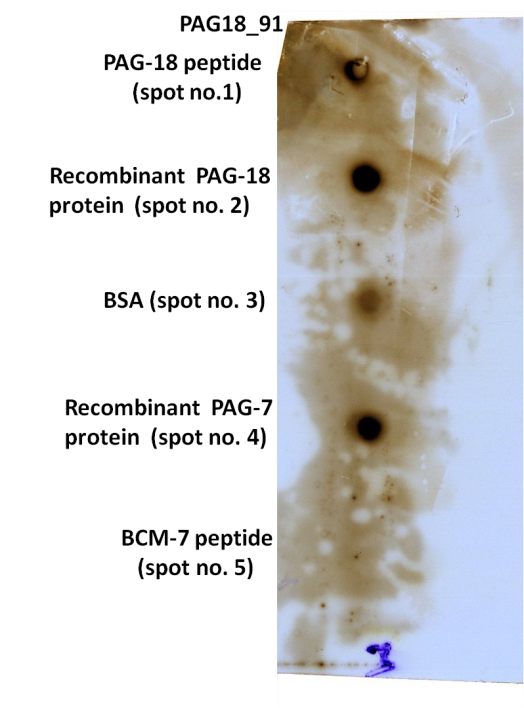

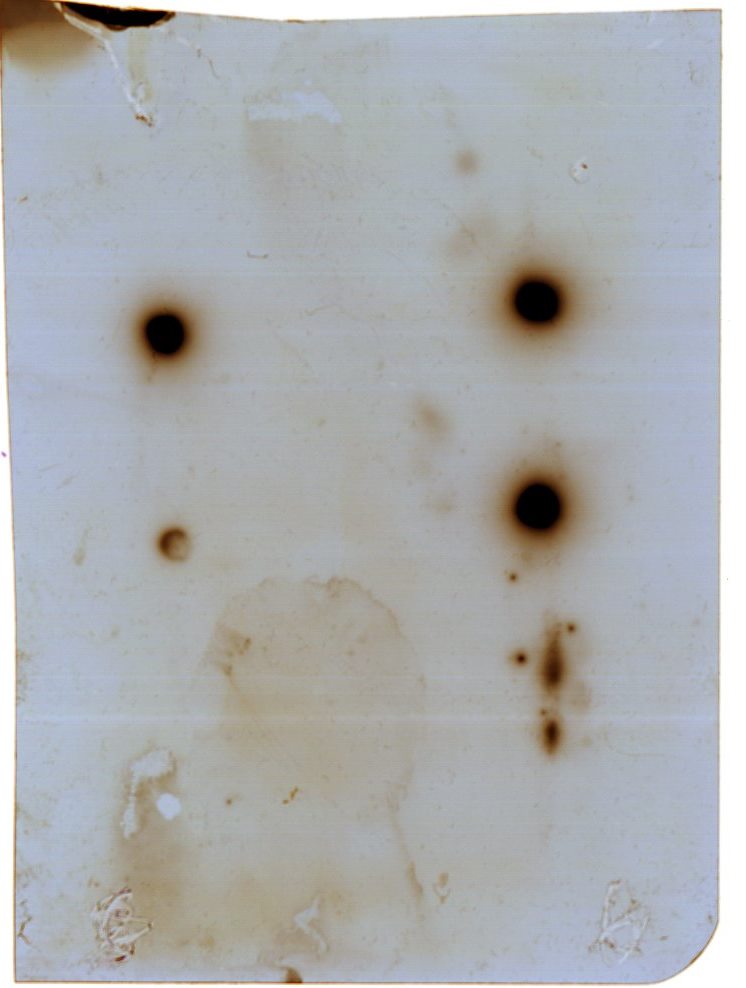

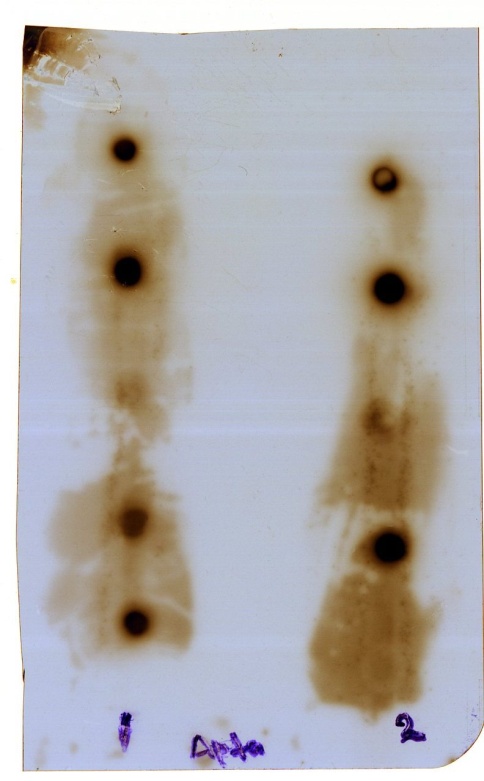

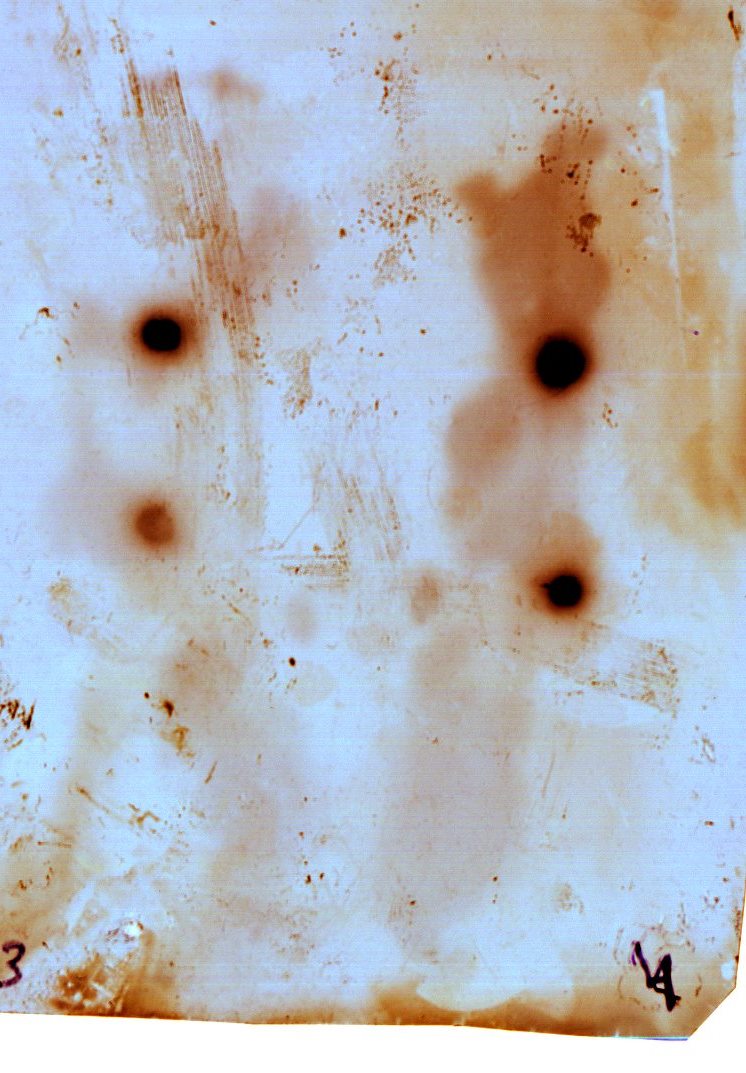


**Fig. 15a Detection of recombinant PAG-7 protein and its derived peptide by aptamer named PAG7_47**

**Fig. 14a Detection of recombinant PAG-18 protein and its derived peptide by aptamer named PAG18_91**

**Interaction study of Seq. PAG18_91 with recombinant PAG-18 protein and its derived peptide**

**Fig. 14b Detection of PAG protein in pregnant sample of animal by aptamer named PAG18_91**

**Fig. 15b Detection of PAG protein in pregnant sample of animal by aptamer named PAG7_47**

**PAG18 peptide (PAG18_91)**

**Recombinant PAG-18 protein (PAG18_91)**

**BSA (C)**

**42 days (PAG 7_47)**

**0 day (PAG 7_47)**

**BSA (C)**

**42 days (PAG7_57)**

**0 day (PAG7_57)**

**BSA (C)**

**42 days (PAG18_91)**

**0 day (PAG18_91)**

**BSA (C)**

**ECL result**

**Fig. 14 & 15 Detection of PAG-18 and 7 proteins by aptamers in ECL & DAB coloring agents**

**PAG-18 peptide (PAG 18_91)**

**PAG-18 recombinant protein (PAG 18_91)**

**BSA (C)**

**Fig. 14**
