# Supplementary material for: Aptamers based sensing of pregnancy associated glycoproteins (PAG) of bovine for early pregnancy detection
Source: Sci Rep. 2021 Dec 1;11:23193. doi: 10.1038/s41598-021-02551-1 (PMC8636505; doi:10.1038/s41598-021-02551-1)
Supplement: Supplementary file 2 — Supplementary Information 2. [file 41598_2021_2551_MOESM2_ESM.pdf]

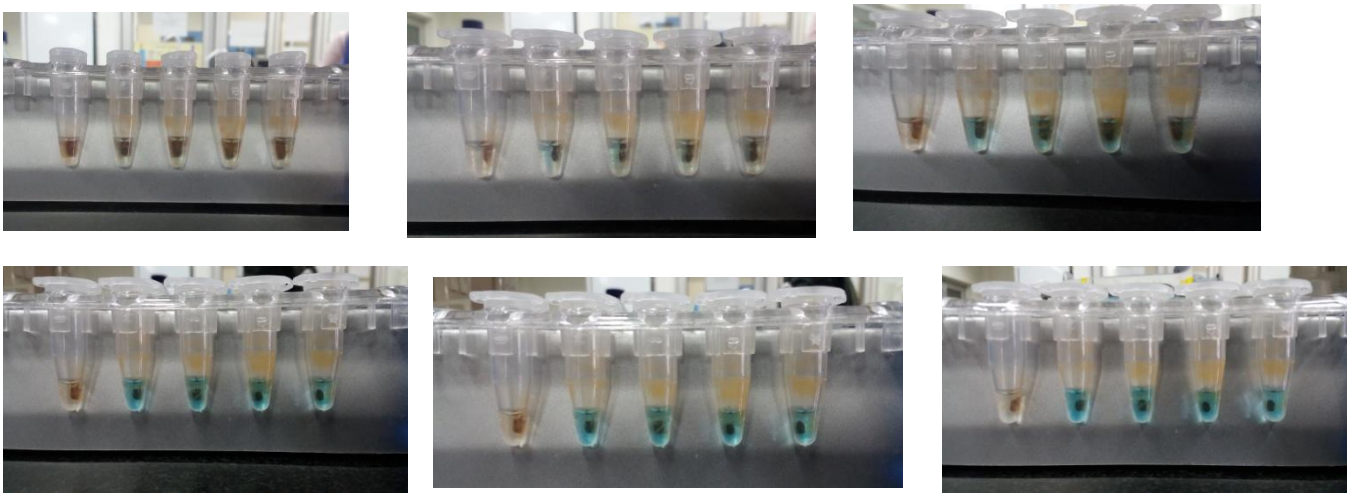

Fig. 2

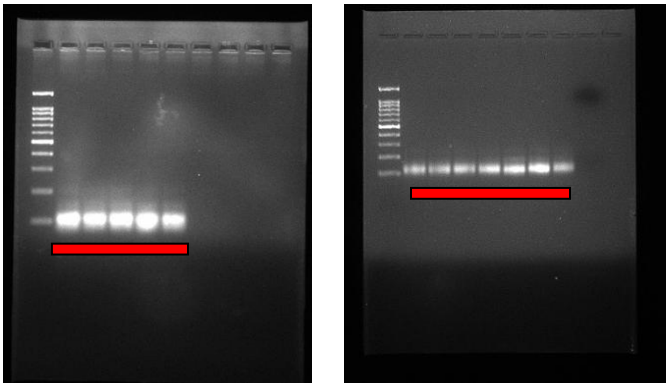

Fig. 3

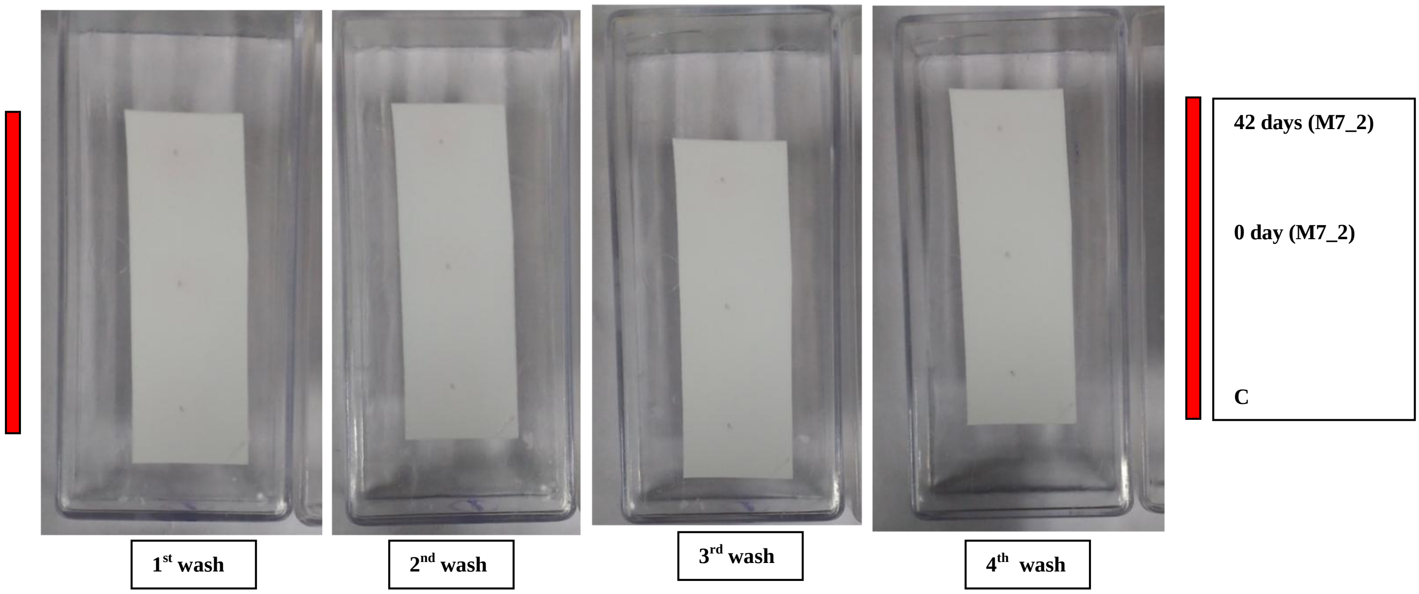

Fig. 6

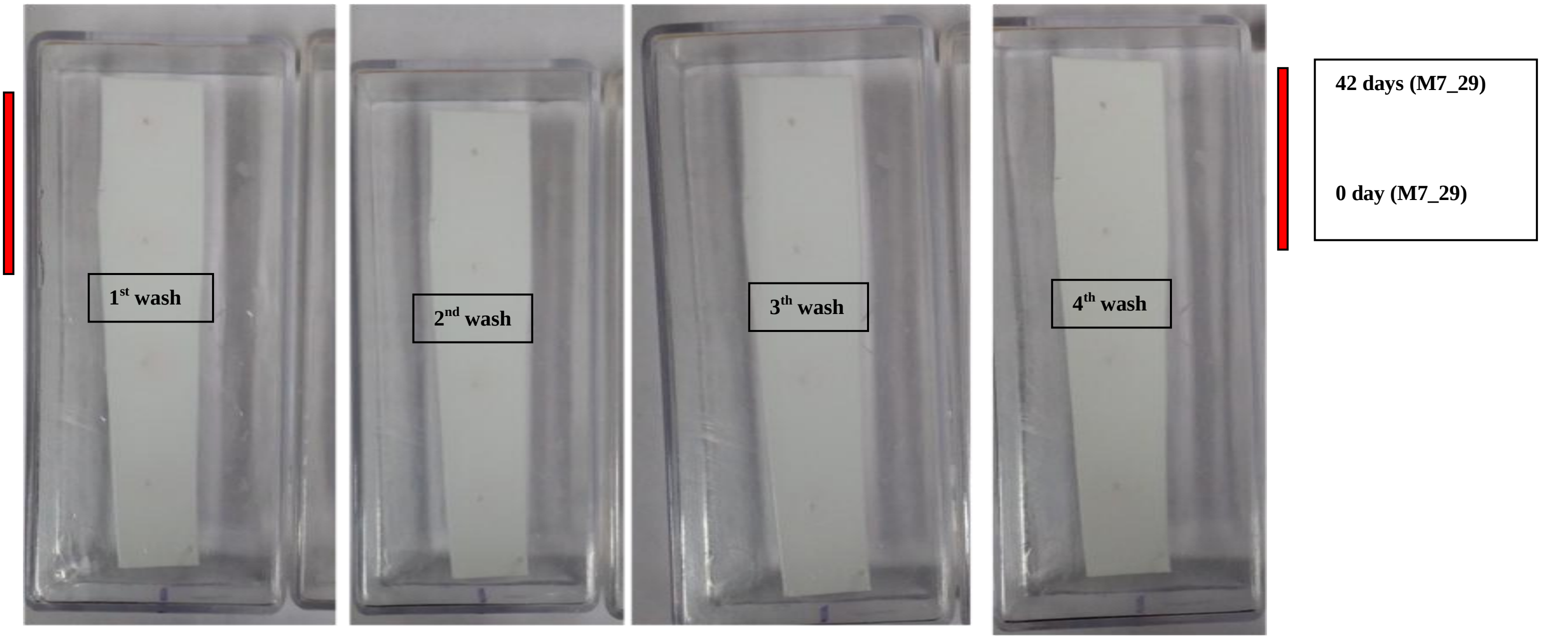

Fig. 7

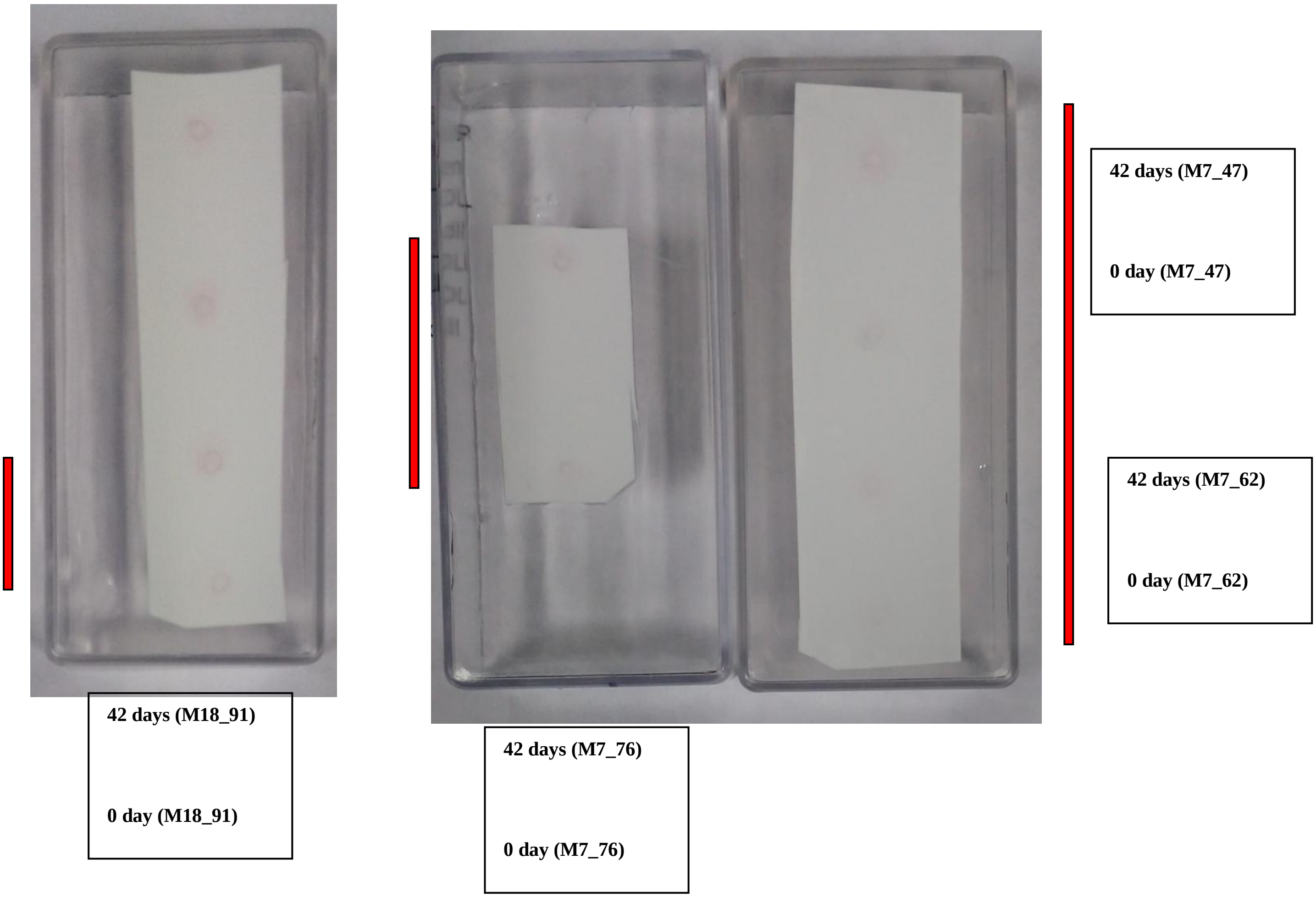
42 days (M18_91)
0 day (M18_91)
42 days (M7_76)
0 day (M7_76)
42 days (M7_47)
0 day (M7_47)
42 days (M7_62)
0 day (M7_62)

Fig. 8

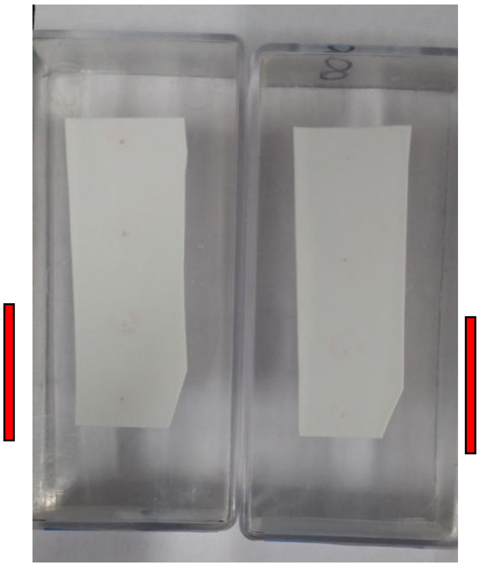

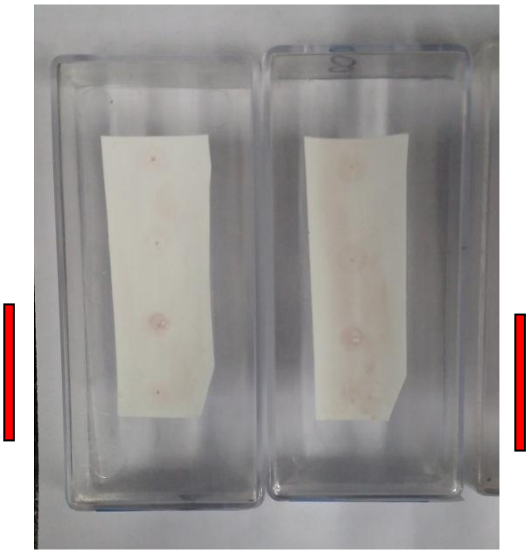

100 days (M18_21)	100 days (M7_29)	Ag^+ enhancement (M18_21)	Ag^+ enhancement (M7_29)
1 day (M18_21)	1 day (M7_29)		

Fig. 9

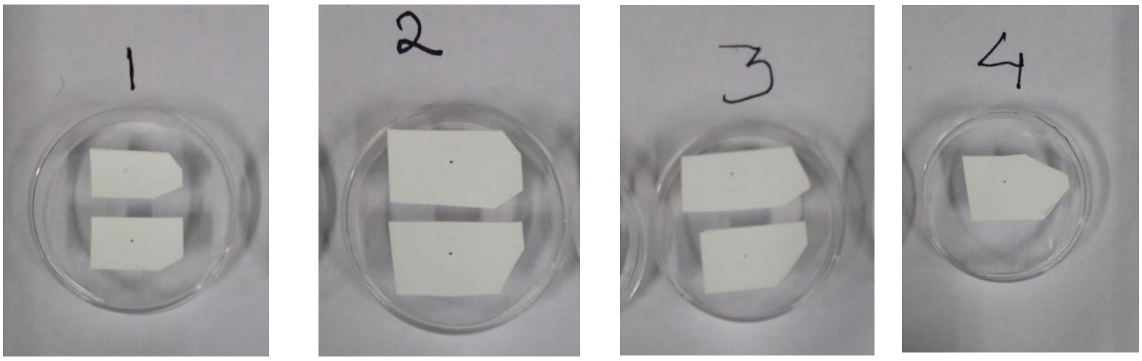

Incubation time	5	10	20	20(c)

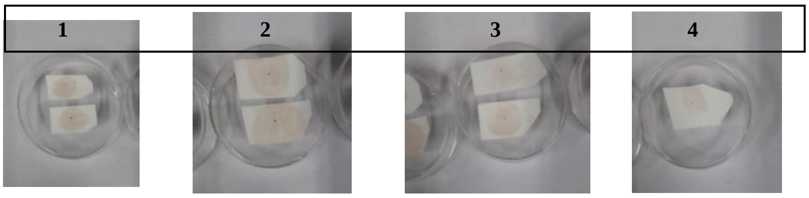

Ag^{+} enhancement

Fig. 11

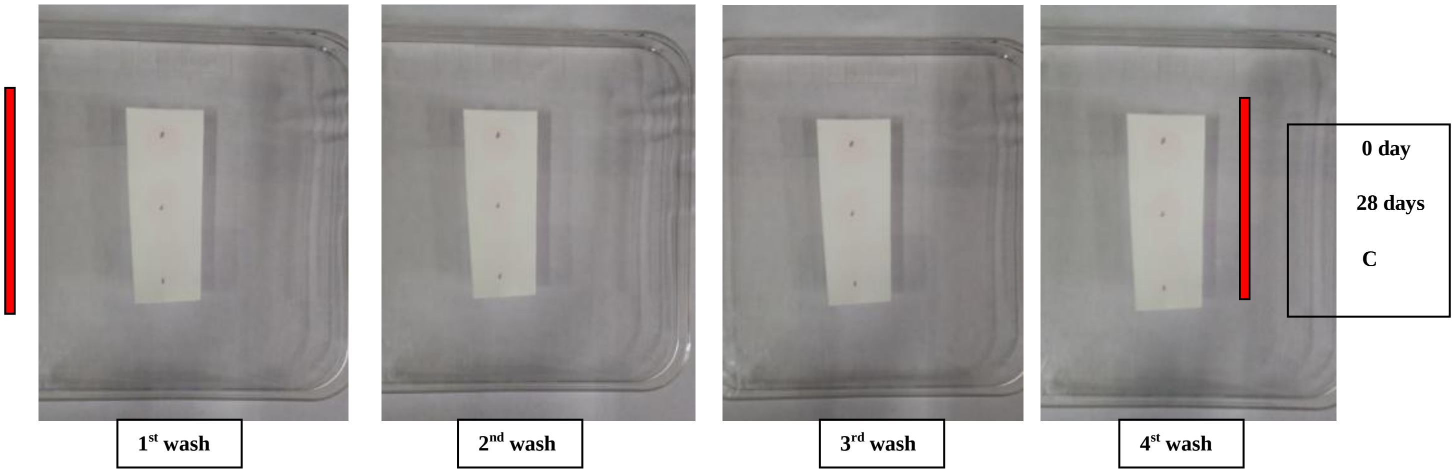

Fig. 12

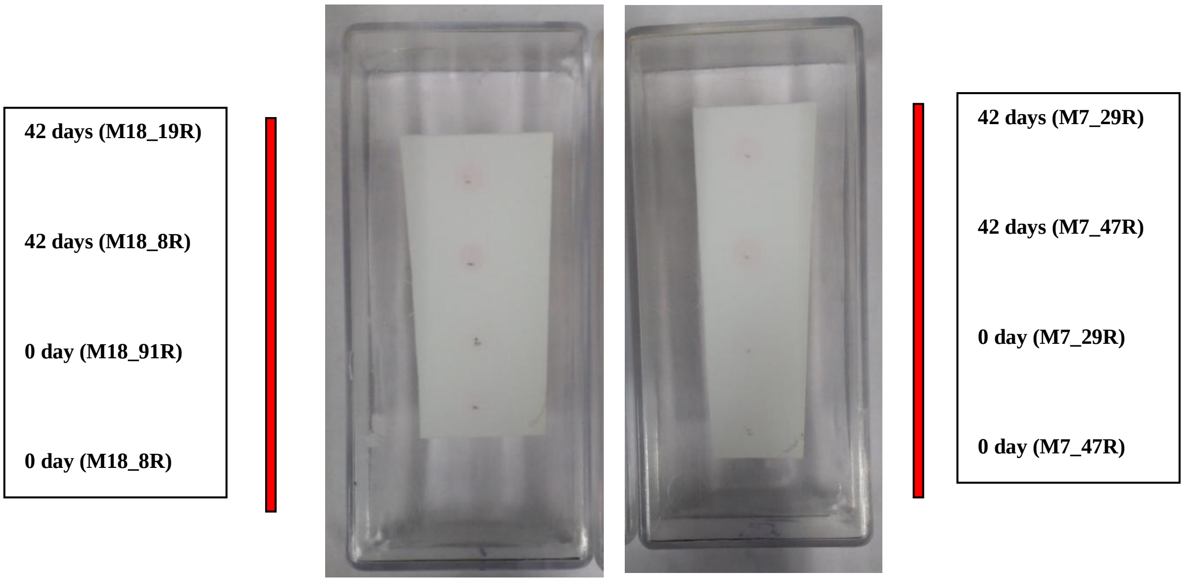
42 days (M18_19R)
42 days (M18_8R)
0 day (M18_91R)
0 day (M18_8R)
42 days (M7_29R)
42 days (M7_47R)
0 day (M7_29R)
0 day (M7_47R)

Fig. 13

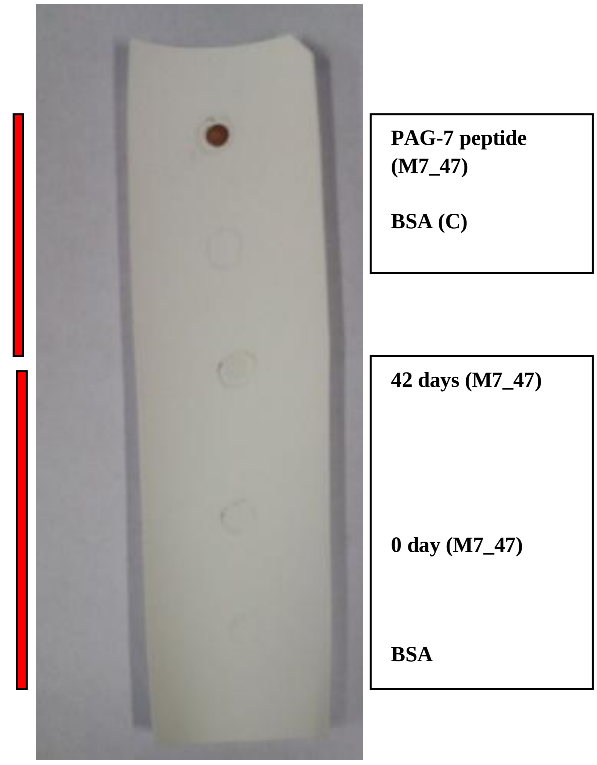

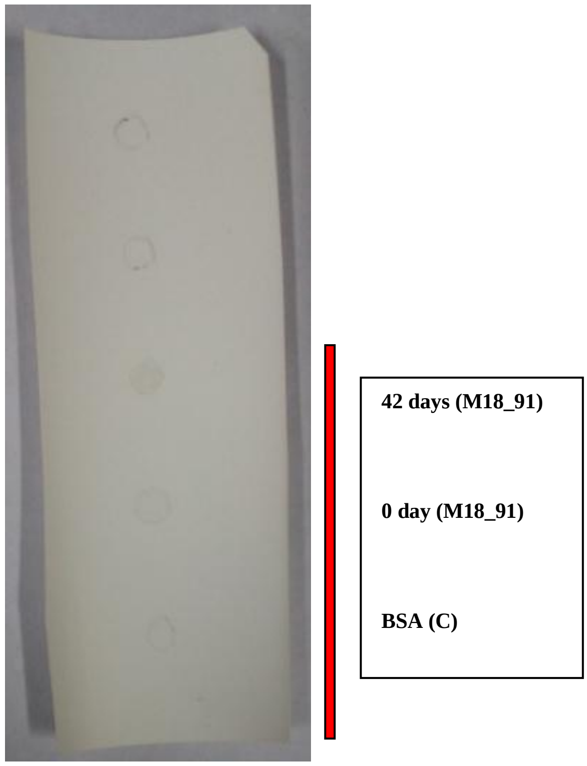

DAB result

Fig. 15

Fig. 14

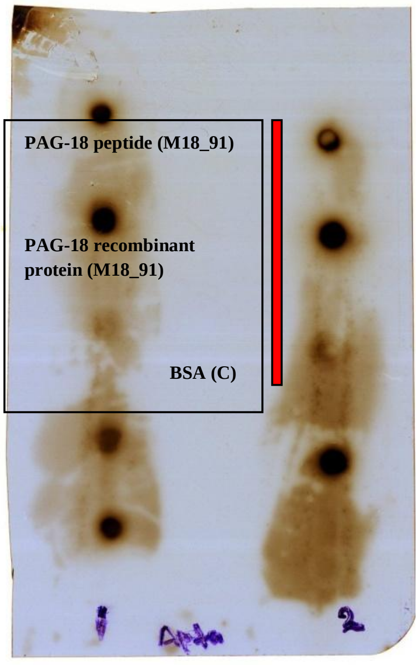

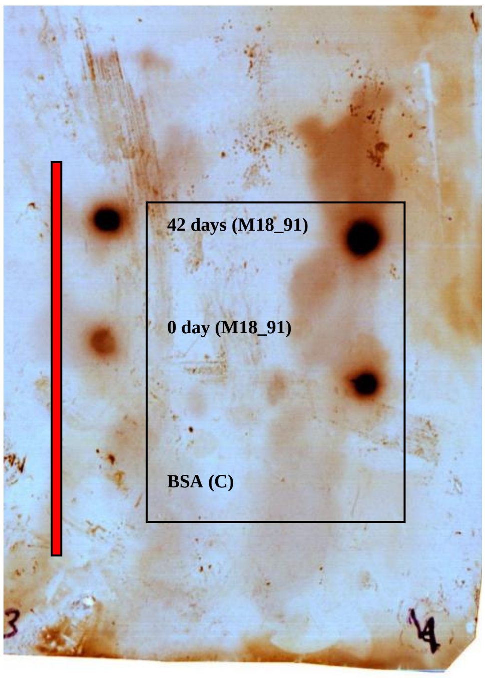

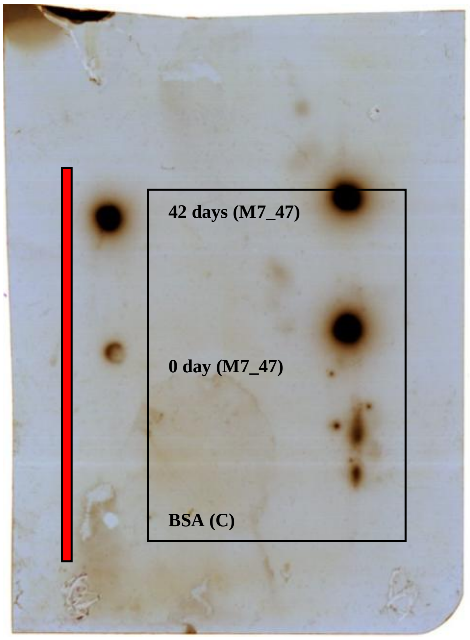

ECL result

Fig. 14

Fig. 14

Fig. 15
